# Characterization of a Proposed *Dichorhavirus* Associated with the Citrus Leprosis Disease and Analysis of the Host Response

**DOI:** 10.3390/v6072602

**Published:** 2014-07-07

**Authors:** José Luis Cruz-Jaramillo, Roberto Ruiz-Medrano, Lourdes Rojas-Morales, José Abel López-Buenfil, Oscar Morales-Galván, Claudio Chavarín-Palacio, José Abrahán Ramírez-Pool, Beatriz Xoconostle-Cázares

**Affiliations:** 1 Departamento de Biotecnología y Bioingeniería, Centro de Investigación y de Estudios Avanzados del Instituto Politécnico Nacional Av. IPN 2508, Zacatenco 07360, México D.F., México; E-Mails: lcruz@cinvestav.mx (J.L.C.-J.); rmedrano@cinvestav.mx (R.R.-M.); abel.lopez@senasica.gob.mx (J.A.L.-B.); jarp1985@hotmail.com (J.A.R.-P.); 2 LaNSE, Centro de Investigación y de Estudios Avanzados del IPN Av. IPN 2508, Zacatenco 07360, México D.F., México; E-Mail: mlrojas@cinvestav.mx; 3 Servicio Nacional de Sanidad Inocuidad y Calidad Agroalimentaria, Guillermo Pérez Valenzuela 127, Coyoacán 04100, México D.F., México; E-Mails: oscar.morales@senasica.gob.mx (O.M.-G.); cchavap@gmail.com (C.C.-P.)

**Keywords:** citrus, plant disease, *Dichorhavirus*, deep-sequencing, plant defense

## Abstract

The causal agents of Citrus leprosis are viruses; however, extant diagnostic methods to identify them have failed to detect known viruses in orange, mandarin, lime and bitter orange trees with severe leprosis symptoms in Mexico, an important citrus producer. Using high throughput sequencing, a virus associated with citrus leprosis was identified, belonging to the proposed *Dichorhavirus* genus. The virus was termed Citrus Necrotic Spot Virus (CNSV) and contains two negative-strand RNA components; virions accumulate in the cytoplasm and are associated with plasmodesmata—channels interconnecting neighboring cells—suggesting a mode of spread within the plant. The present study provides insights into the nature of this pathogen and the corresponding plant response, which is likely similar to other pathogens that do not spread systemically in plants.

## 1. Introduction

Citrus leprosis is a viral disease with important economic implications that is rapidly spreading in the New World. It has been reported throughout America, although not observed in the last four decades in the U.S., causing severe damage in different citrus cultivars [1,2,3,4]. Besides the appearance of symptoms, which result in lower fruit quality, leprosis negatively impacts productivity, which is affected by a decrease in foliar area, premature leaf abscission and branch death. This disease is widespread from Argentina to Costa Rica; recently, it has been detected in Chiapas, Southern Mexico, and different viruses appear to be associated with it [5]. However, the precise identity of the associated pathogen remains to be determined, at least in Mexico. More recently, *Citrus* spp. plants (sweet orange, mandarin and grapefruit) showing leprosis symptoms have been observed in the state of Jalisco, central-western Mexico, indicating the spread of the disease. Thus, the complete characterization of this pathogen is necessary to design plausible control strategies.

Leprosis symptoms include local, albeit severe, necrotic symptoms in infected leaves, as well as in fruits, in addition to corked stems. The most notable cytopathic effects are the appearance of electron-dense inclusion bodies in the cytoplasm or in the nucleus [4]. The cytoplasmic leprosis is the more prevalent. Viruses that cause leprosis symptoms in various citrus cultivars have been identified [6,7,8,9,10,11]. The most widely distributed is the *Citrus leprosis virus* cytoplasmic type, CiLV-C, which harbors two single-stranded positive stranded RNA components, both of which are capped and polyadenylated [7]. This virus is the type member of the *Cilevirus* genus and is the etiological agent of cytoplasmic leprosis [7]. More viruses have been identified causing similar diseases in citrus, such as the newly discovered *Citrus leprosis virus* cytoplasmic type 2 (CiLV-C2) in Colombia, which is related to other Cileviruses; it consists of two single-stranded (ss) positive-stranded RNA components. While its genomic organization is similar to CiLV-C, it possesses an additional open reading frame in the RNA2 component [11]. Another related virus infecting *Citrus volkameriana*, *Hibiscus green spot virus* (HGSV), harbors three ssRNA (+) components, displaying a similar genomic organization to CiLV-C2 [9]. Phylogenetic analyses of these viruses indicate that they are type members of new genera. Furthermore, all these viruses have in common their mode of transmission by mites of the *Brevipalpus* genus (Acari: Tenuipalpidae) [6] and are not systemic in nature [6,7,8,9,10,11,12,13,14]. Seventy-one species of false spider mites representing five genera (*Pseudoleptus*, *Aegyptobia*, *Tenuipalpus*, *Brevipalpus* and *Priscapalpus*) in Mexico have been described [14]. Citrus leprosis can also be transmitted mechanically [15]. In all, this evidence suggests that different viruses could elicit similar responses from their hosts and, thus, symptomatology.

There are several members of the *Rhabdoviridae* and the *Cilevirus* genus among viruses transmitted by spider mites. These are related in terms of their genome organization, overall sequence similarity, virion morphology (enveloped bacilliform structures *vs.* bullet-shaped enveloped virions) and cytopathic effects on their hosts [7,13]. However, there are important differences, namely that the genome of rhabdoviruses consists of monopartite negative-ssRNA. Additional unrelated viruses causing leprosis symptoms belong to the *Mandarivirus* genus, which are related to *Potexvirus* and, thus, display a monopartite ssRNA genome of a positive polarity [8]. In all cases, the cytopathic effects in hosts are quite similar.

Rhabdovirus particles can accumulate either in the cytoplasm or in the nucleus, forming large electron-dense inclusion bodies, or viroplasms, where the replication of the virus occurs [16,17]. The genome assembly of a potential CiLV-N in Citrus trees with high similarity to *Orchid Fleck Virus* (OFV) was described [10].

As mentioned before, citrus leprosis has been detected in Mexico; given that different viruses may cause similar symptomatology in citrus, it was not clear whether the causal agent was CiLV (cytoplasmic or nuclear) or a hitherto unknown virus. ELISA and RT-PCR performed on symptomatic leaves from infected citrus in Jalisco and Chiapas failed to detect quarantine virus or other known pathogens in Mexico. In order to determine the identity of the pathogen causing the observed symptomatology, a different strategy was devised; RNA was obtained from bitter orange (*C.* × *aurantium*) leaves showing leprosis symptoms and employed for high throughput sequencing (HTS). Novel sequencing technologies have allowed the elucidation of large genomes, and RNA-seq has been used recently to analyze diverse transcriptomes [18]. Combined with bioinformatic analysis, this methodology can be used to compare sets of transcriptomes for two different biological conditions [19,20], as well as for the detection and analysis of low-abundance RNAs, which may be useful for the detection of novel pathogens or symbionts [18]. In the present work, we describe the characterization of a novel virus, termed Citrus necrotic spot virus (CNSV) belonging to a proposed new group, *Dichorhavirus*, associated with citrus leprosis in bitter oranges in Mexico. This virus was found to be similar to *Orchid fleck virus*, as well as to a recently sequenced Citrus Leprosis Virus-Nuclear (CiLV-N) [10] and Coffee ringspot virus (CRSV), the sequences of the potential open reading frames of CRSV, which have been deposited recently in GenBank (Accession Nos. AHH44825.1, AHH44826.1, AHH44827.1, AHH44828.1, AHH44829.1 and AHH44830.1). The cytoplasmic localization, as well as the non-coding regions of this virus suggest that it is different from other Dichorhaviruses.

## 2. Materials and Methods

### 2.1. Plant Material

Leaves and fruits with necrotic and/or chlorotic spots were collected from 67 citrus cultivars *Citrus* × *aurantium* (bitter orange), lime (*Citrus sinensis*) in temperate regions of Mexico, mostly in Guadalajara, Jalisco, (20.663626° N, 103.375854° W) and Tecpatán (16.99768° N, −93.46194° W) and Ocozocuautla (16°46'0" N, 93°22'0" W), Chiapas. These symptoms were similar to those described on Citrus leprosis-affected trees. The collection of plant material was performed by the Jalisco and Chiapas State Committees for Plant Health (Comité Estatal de Sanidad Vegetal de Jalisco and Comite Estatal de Sanidad Vegetal de Chiapas, respectively) [21,22], which are government agencies authorized by the Mexican Department of Agriculture for this purpose. False spider mites were observed in infected citrus tissue. All plant samples were shipped and stored at −80 °C until used. Field studies did not involve endangered or protected species.

### 2.2. RNA Isolation for High Throughput Sequencing

Total RNA from *Citrus* × *aurantium* asymptomatic and symptomatic leaves obtained from ten different trees were isolated using the RNeasy kit (Qiagen, Hilden, NRW, Germany); genomic DNA was treated with DNaseI (Invitrogen, Carlsbad, CA, USA) and sent for the Illumina Whole Transcriptome Shotgun Sequencing platform (RNA-seq) to Otogenetics^©^ Corporation (Atlanta, GA, USA). DNA sequencing was performed from cDNA, synthesized from Citrus poly(A+) RNA, as requested from the sequencing service. Two replicates of RNA-seq sets for symptomatic and asymptomatic samples were delivered. Each RNA-seq set consisted of 7.5 × 10^7^ reads and was 74 bases long, with a total of 15.0 × 10^7^ for each biological condition.

### 2.3. Bioinformatic Analysis

A Bio-Linux v7.0 workstation was employed to analyze the raw data of RNA-seq [23,24,25,26], comprised of 15 million short reads of 74 bases in length, in files in the FastQ format [27]. Quality scores were verified with FastQC, which were acceptable. Raw data were retrieved from the sequencing service and compared against the reference genome of *Citrus × clementina* downloaded from the Phytozome V9.0 database [25]. The reference genome was indexed with the Bowtie-build indexer tool from the Bowtie package in order to align RNA-seq reads to the *C. × clementina* genome [27]. Modified parameters consisting of minor gap penalizations for TopHat 2 were used, which yielded two sets of files, the first set of reads of which had homology with the reference genome and the second set of which was not aligned with the *C. × clementina* genome. The homologous reads were analyzed to identify differential gene expression for both asymptomatic and symptomatic tissue [24,28,29,30,31]. The differential expression was performed with the Cufflinks, Cuffmerge and Cuffdiff default parameters [24]. A reference annotation file (GFF3 format) was downloaded, as well, for *C. × clementina*, from the Phytozome database. Unaccepted reads that had no homology with the reference genome were first analyzed with BLAST tools and, later, were overlapped with the assembly tool in the CLC Genomics Workbench version 6.0 (CLC bio, Aarhus, J, Denmark). Several contigs were retrieved, and a BLAST was performed against the GenBank database [32]. The BLAST provided information of which contigs could be part of viral genomic components. The further reassembly of previous contigs was performed using different assembly programs, including Mira v3.9.10 [33]. Potential open reading frames (ORF) were obtained using Artemis [34], and the annotation for the complete sequences was submitted to NCBI GenBank, Accession Nos. KF198064 for CNSV RNA1 and KF198065 for CNSV RNA2.

### 2.4. Phylogenetic Analysis

Amino acid sequences from the *Rhabdovirus* genus were retrieved from the NCBI non-redundant protein database (Table S1). Alignments for sequences of Nucleocapsid (N) and RNA-dependent RNA polymerase (L) were made with Seaview [35] and ClustalX-2 [36]. Each alignment was optimized for L sequences (the ClustalW algorithm using the PAM350 substitution matrix, an iteration after each alignment) and N sequences (the multiple sequence comparison by log-expectation algorithm, known as MUSCLE with 6 maximum iterations). The best-fit substitution models for the alignments, as well as the amino acid frequencies (+F), the proportion of invariable sites (+I), the substitution rate categories and the gamma shape (+G) parameters were selected according to ProtTest 3.0 [37] for L protein sequence alignment (RtREV + I + G + F) and N protein sequence alignment (LG + I + G + F). Phylogenetic reconstruction was performed using the PHYML platform [38,39,40], and 100 bootstraps for branch support was selected. Rooted trees were plotted with FigTree v1.4.0 [41].

### 2.5. Differential Expression Analysis

Differential expression from both infected and asymptomatic samples was determined as described before following the Tuxedo Protocol [24,26]. Transcript abundance was quantified in fragments per kilobase of exon per million mapped (FPKM) using the Cufflinks software package. A combined annotation file for both conditions was merged with the reference annotation consisting of the *C. × clementina* GFF3 file (Cuffmerge). The log2 fold change for both conditions was used to cutoff for up- or down-regulated genes. *P*-values and E-values were tested to identify significant differences between the two conditions using Cufflinks [24,42]. Genes differentially expressed were categorized by known function or possible function. The gene annotation of differentially expressed transcripts was carried out first, by searching for their gene ID sequence and later by submitting to the automatic annotation server, Blast2GO [43,44]. Annotations consisted of automatic search for sequence homology, Gene Ontology annotation, as well as signature protein InterProScan collection. In order to graphically display differential gene expression, MapMan software was employed [45]. For this purpose, *Citrus × aurantium* up- and down-regulated genes were employed to identify orthologous *Arabidopsis thaliana* genes, considering the annotation provided by Phytozome V9.1.

### 2.6. RT-PCR Analysis

Total RNA purification was performed using the method reported by Logemann *et al.* with minor modifications [46]. The resulting RNA was then cleaned up using a commercial system, following the manufacturer’s instructions (Qiagen, Santa Clarita, CA, USA). Once the identification of candidate viral contigs was completed, primers were designed to detect both RNA1 and RNA2 genomic components: for RNA1, CNSV1F (5'-GCTAATCCAAGTGAGATCGATTACATGAC-3') and CNSV1R (5'-GCTGTCCTGCCTTGTCTTGATGTCCG-3'), the target sequence is located between 69 and 428 nt in RNA1, synthesizing a 360-bp fragment; for RNA2, CNSV2F (5'-TCCCGTCCGGACTTTCACTGTCCATAAGT-3') and CNSV2R (5'-GATGTTTGGCGAAAGGTCCATGTGTGGAT-3'), located between 836 and 1315 nt in RNA2, synthesizing a 480 bp fragment.

A One Step RT-PCR assay was performed for the molecular detection of CNSV using the SuperScript™ III One-Step RT-PCR System with the Platinum^®^ Taq High Fidelity Kit (Invitrogen, Carlsbad, CA, USA). The total RNA extracted from asymptomatic *C. × aurantium* leaves was used as the negative control. As a template for the assay, 100 ng of Total RNA samples from infected tissue were used. The RT-PCR One-Step reaction was performed following the manufacturer’s instructions. The primer mixtures used were CNSV1F/CNSV1R and CNSV2F/CNSV2R, expecting 360-bp and 480-bp amplicons, respectively. A 12.5-µL RT-PCR reaction was prepared for each RNA sample and each primer mixture with the following program: 1 cycle at 50 °C, 30 min for cDNA synthesis; 1 cycle at 94 °C, 5 min for Platinum^®^ Taq activation; 40 cycles at 94 °C for 35 s, 62 °C for 20 s and 72 °C for 45 s; and 1 cycle at 72 °C for 5 min. Amplification products were resolved in 1.0% w/v agarose gel stained with ethidium bromide and visualized under UV light.

### 2.7. Validation of Differentially Gene Expression through Quantitative RT-PCR

Differential gene expression for both symptomatic and asymptomatic samples was validated through qRT-PCR for the following transcripts encoding for: germin-like protein, thaumatin-like protein, PR-3 class IV chitinase, proline-rich protein 4-like, aquaporin tip-2 like and housekeeping cytochrome c oxidase. Forward and reverse oligonucleotides were designed in order to amplify a segment of each transcript sequences as described in Table S2. The quantitative RT-PCR reaction was set up with the KAPA SYBR^®^ FAST One-Step qRT-PCR Universal Kit (KAPA Biosystems, Boston, MA, USA) following the manufacturer’s instruction and set up on a Rotor Gene 6000^®^ real-time amplification system (Corbett Life Science, Hilden, Germany), using the following program: 1 cycle at 42 °C, 10 min for cDNA synthesis; 1 cycle at 95 °C, 5 min for Taq activation; 45 cycles at 95 °C for 5 s, 60 °C for 30 s and 72 °C for 5 s; and a melt profile from 50–99 °C, rising 1 °C each 5 s. Absolute expression levels were quantified by constructing a standard curve using cDNA dilutions of each gene, and statistical significance was tested using the F distribution.

### 2.8. Transmission Electron Microscopy

Leaf tissues with chlorotic and necrotic spots were collected from symptomatic trees and processed as follows: tissue was cut in 1-mm^2^ squares and transferred to microtubes containing 2% glutaraldehyde in 1× PBS for 2 h. The fixative was removed, and the tissue was rinsed twice with 1× PBS. Postfixation was carried out with 1% osmium tetroxide in the same buffer for 12 h. Samples were dehydrated in ethanol series from 20% to 100% and incubated in propylene oxide, to finally be embedded in Araldite 502 resin (Electron Microscopy Sciences, Hatfield, PA, USA). Ultrathin sections (Ultracut E Reichert Jung, Reichert Technologies, Depew, NY, USA) were placed on 200-mesh Formvar-coated copper grids. Sections were then contrasted with 2% uranyl acetate, followed by lead citrate and then examined with a JEOL 2000EX transmission electron microscope (JEOL, Kyoto, Japan) at 80 KV.

## 3. Results

### 3.1. Citrus Leprosis Symptomatology in Citrus Plants in Mexico

**Figure 1:**
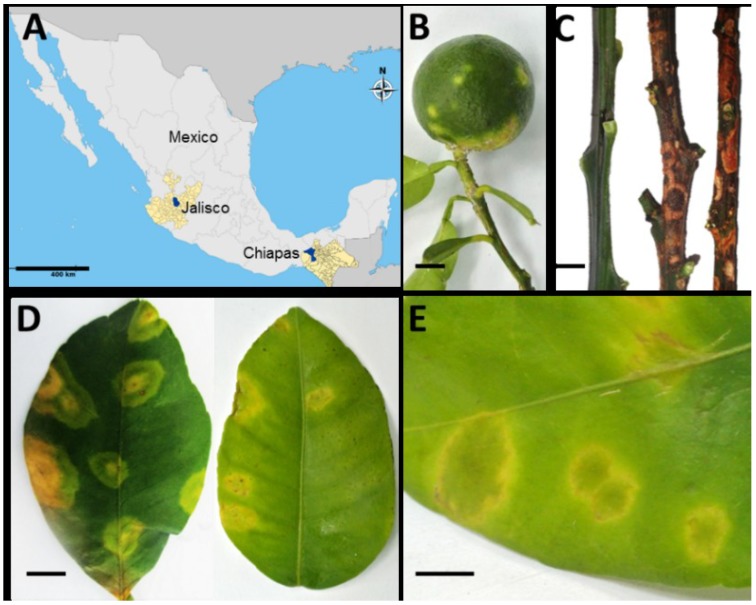
Symptoms of leprosis in bitter orange in Mexico. (**A**) Plant tissue was collected in the urban zones of Guadalajara, Jalisco, and Tecpatán and Ocozocuautla, Chiapas; shown in blue in the map. (**B**) Typical symptoms found in bitter orange (*C.* × *aurantium*) fruit, including chlorotic spots, which, in later stages of the disease, appear brown. (**C**) A healthy stem is shown on the left; a corking and brownish stem can be observed in infected tissue. (**D**) Similar chlorotic spots were observed in leaves of infected *C.* × *aurantium*, which are distributed asymmetrically on the leaf surface. Leaves also display rugosity and asymmetric development. (**E**) Close-up of an infected leaf.

Leaves, fruits and stems from 67 symptomatic citrus trees with leprosis lesions were collected in conurbated zones of Guadalajara in the state of Jalisco and Tecpatán and Ocozocuautla, Chiapas, Mexico (Figure 1A). Guadalajara City is located at 1552 m above the sea level (masl) with temperate weather, while the cities of Tecpatán and Ocozocuautla are located at 310 masl with tropical weather. Fruits exhibited chlorotic and necrotic spots in both immature and mature fruits (Figure 1B), while infected stems are corked with brownish spots (Figure 1C, center and right stems), in contrast with healthy green stems (Figure 1C, left stem). Leaf lesions consisted of chlorotic areas, with necrotic borders, asymmetrically located on the leaves (Figure 1D,E). Defoliation was a recurrent symptom observed in trees affected by this disease.

### 3.2. A Virus Belonging to the Proposed *Dichorhavirus* Genus is Associated with Citrus Leprosis Symptoms in Mexico

The agent causing leprosis-like symptoms in citrus cultivars in Mexico had not been identified, and since extant diagnostic methods yielded negative results for bacteria and known viruses, it was hypothesized that an unidentified virus causes this disease. Samples from sweet orange, bitter orange and grapefruit showing such symptoms were tested for CiLV-C and HGSV, which yielded negative results (Figure S1). Thus, in order to gain insight into the etiology of this disease, high throughput sequencing of RNA from symptomatic leaves of sour orange (*Citrus × aurantium*) was carried out. This plant is a hybrid of *C. maxima* and *C. reticulata* and is widely grown in Mexico, and in Texas and Florida in the U.S. [47]. Since *Citrus* × *clementina* (clementine, which is a *Citrus* × *aurantium* × *C. reticulata* hybrid) is the closest relative of *Citrus* × *aurantium*, its genome was used as a scaffold to determine the transcriptome of symptomatic leaf tissue. The 3 × 10^7^ reads were obtained and compared to the *C. × clementina* genome database, as well as to another RNA sample from asymptomatic leaves from *C.* × *aurantium*. Approximately 1.2 × 10^4^ reads did not match the *C. × clementina* nor *C. sinensis* genomes in symptomatic samples. The coverage of the viral genome by the contigs was redundant (Table 1, Figure S2). No other contigs absent from the *C. × aurantium* or *C. sinensis* genome databases were found, with the exception of a bacteriophage highly similar to φX174. Additionally, only 3 reads for CNSV RNA1 and 4 for CNSV RNA2 were found in the asymptomatic tissue used as a control; these reads showed close similarity to OFV and to a recently reported CiLV-N [10]. The reads were assembled into two genomic RNA components that are indeed similar to OFV and CiLV-N (90% overall homology). (Figure S3).

**Table 1:** Reads of symptomatic and asymptomatic samples.

Total no. of reads for symptomatic tissue	7,737,481
Total no. of reads for asymptomatic tissue	7,291,897
Total	15,029,378
Total no. of reads for RNA1 (6087 nt)	12,083
Average read	196.87 nt
Total no. of reads for RNA2 (6015 nt)	12,079
Average read	199.39 nt
Total length of coverage of cDNA reads for symptomatic tissue	2.01 Gb
Total length of coverage of cDNA reads for asymptomatic tissue	1.90 Gb

**Figure 2:**
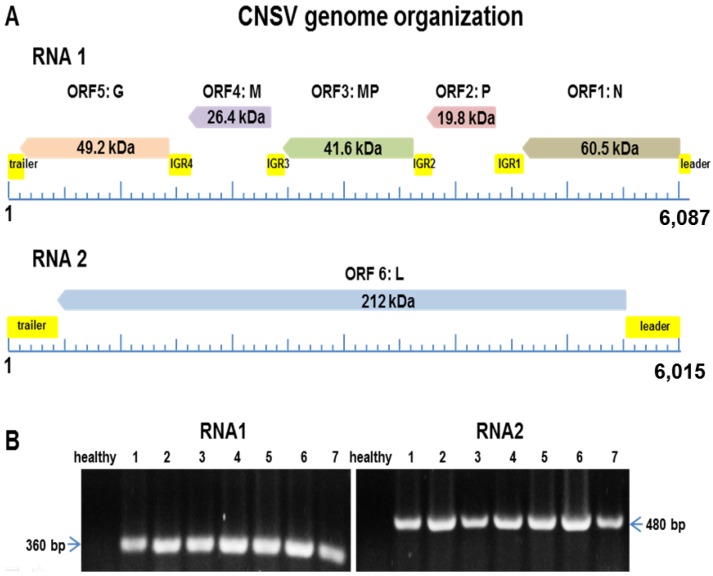
Map of Citrus Necrotic Spot Virus (CNSV) genome components and putative open reading frames in both RNA1 and two components. (**A**) Leader and trailer RNAs and intergenic regions (IGR) are also depicted. N, nucleocapsid protein; P, phosphoprotein; MP, movement protein; M, matrix protein; G, glycoprotein; L, RNA-dependent RNA polymerase. (**B**) RT-PCR from representative *C.* × *aurantium* leaf samples showing leprosis symptoms. (**Left**) The lane marked as “healthy” is non-symptomatic tissue and Lane 1 to 7: independent samples. The left panel is the detection of the RNA1 component. (**Right**) The detection of RNA2.

The assembled viral genome consists of two ssRNA components, RNA1, 6495 nt in length, and RNA2, 6018 nt in length. RNA1 harbors four open reading frames, potentially coding for the nucleocapsid protein (N) (ORF1, 450 aa in length, 60.5 kDa); a putative phosphoprotein (P) that also forms part of the nucleocapsid (ORF2, 237 aa, 19.8 kDa); a putative movement protein (MP) (ORF3, 370 aa, 41.6 kDa), which has been functionally characterized in other plant rhabdoviruses [48,49]; the putative matrix protein (M) involved in maintaining virion shape (ORF4, 183 aa, 26.4 kDa); and a potential glycoprotein (G) of unknown function (ORF5, 450 aa, 49.2 kDa) (Figure 2A). On the other hand, RNA2 consists of a single ORF (ORF6), potentially coding for the RNA-dependent RNA polymerase (L), which is an 853-aa protein with a theoretical MW of 212 kDa (Figure 2B). The putative function of each ORF was deduced on the basis of similarity to the OFV RNA1 and 2 sequences [50]. A comparison between OFV, CiLV-N and CNSV shows a highly conserved genome organization. All ORFs from these viruses are highly similar; however, both leader and trailer RNA sequences in the two components do differ between CNSV and OFV (Figure S3). The CNSV RNA1 leader displays a similarity of 89% with OFV, while CiLV-N shows 96% homology to OFV. Because of the striking similarity between CiLV-N and OFV sequences, they appear to be variants of the same strain. Both intergenic regions (IGR) and trailer RNA are highly conserved in the three compared sequences. A comparison of RNA1 and RNA2 components (Figure S3) shows differences in both leader and trailer sequences, *i.e.*, there are three deletions in both CILV-N and OFV in RNA1, when compared to CNSV, while intergenic region 1 (IGR1) also harbors a four-base deletion. Of note is the RNA1 trailer region, in which CNSV has the larger sequence: 34 bp more than OFV and 184 bp more than CiLV-N. As for the RNA2 leader, two deletions are again present in OFV and CILV-N (Figure S3), as well as two deletions of four and one bases, respectively, located toward the ORF6 5’ end. Based on these differences, we propose that this could be considered a different strain of OFV and CiLV-N, which was designated provisionally Citrus necrotic spot virus (CNSV).

It must be mentioned that while the assembled sequence of RNA2 displayed negative polarity, as do all rhabdoviruses hitherto described so far, the contigs assembled with Cufflinks corresponding to RNA1 and RNA2 displayed both polarities, suggesting the identification of possible RNA(+) replicative intermediates. Therefore, the ratio of sense *versus* anti-sense sequencing reads was also determined. As indicated by the log-odds (Lods) values of sense to antisense reads, RNA1 was marginally enriched for sense-mapping reads and RNA2 for antisense reads. Based on the genome organization and homology with the most closely related virus (OFV), it is likely that both RNA genome components display negative polarity.

**Figure 3:**
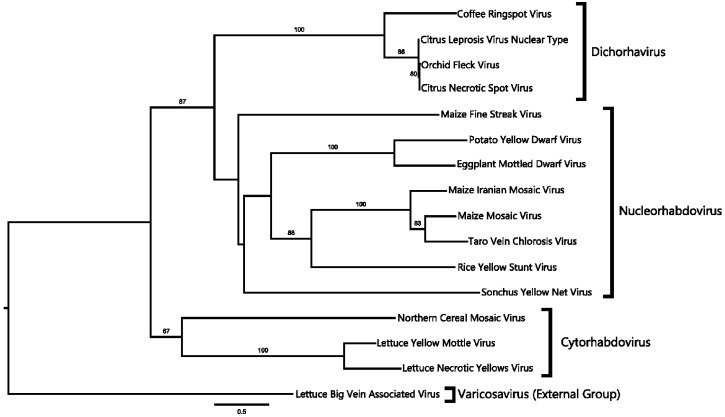
Phylogenetic reconstruction for nucleocapsid sequences. Tree construction is based on the maximum likelihood model with a bootstrap value indicated above supported branches. CNSV and *Orchid Fleck Virus* (OFV) and CiLV-N are grouped in a single branch, marked as Dichorhavirus.

**Figure 4:**
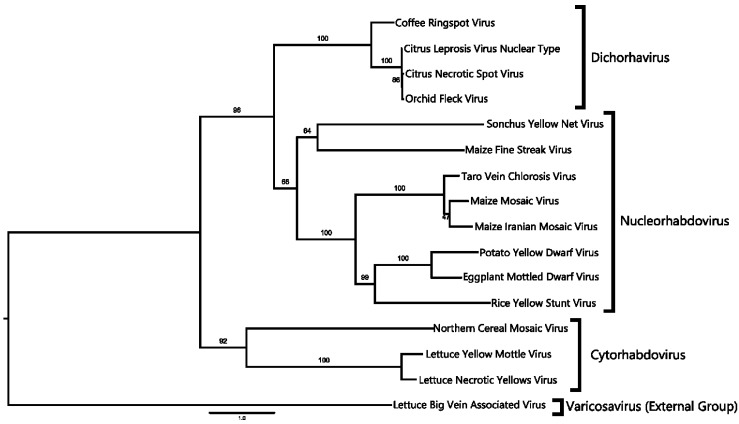
Phylogenetic reconstruction for RNA-dependent RNA polymerase sequences. Tree construction is based on the maximum likelihood model with a bootstrap value indicated above supported branches. The proposed Dichorhaviruses are grouped in a single branch.

The phylogenetic relationship of CNSV to other viruses was determined based on the ORF1 (nucleocapsid protein, N) and ORF6 (RNA-dependent RNA polymerase, L) amino acid sequences, as well as on the full-length genome. Rooted distance trees were constructed with 100 bootstraps. In all cases, CNSV forms a single clade with OFV and CiLV-N and, thus, can be considered a member of the proposed clade, Dichorhavirus ([50]: Figure 3 and Figure 4). This clade, based on different trees, is further located from other plant Rhabdovirus groups. A closer inspection of the tree based on the nucleocapsid protein sequence revealed that CNSV and OFV do not fall within the nucleorhabdovirus clade (Figure 3). Similar results were obtained with the RdRp and whole genome sequences (Figure 4). OFV, CiLV-N and CNSV likewise are grouped in a separate clade outside cytorhabdovirus and nucleorhabdovirus. This clade comprising CNSV, as well as OFV and CiLV-N, correspond to the newly proposed Dichorhavirus genus [6], which includes bipartite, negative-strand RNA plant viruses.

RT-PCR analysis was carried out for 67 leaf samples showing leprosis symptoms from diverse citrus cultivars, including *C.* × *aurantium*, *C. sinensis*, *C. tangerine* and *C. paradisi*. Sixty five samples yielded positive results for both RNA1 and RNA2 CNSV components and failed to detect them in asymptomatic tissue. The analysis of eight samples is shown in Figure 2. Thus, there is a strong correlation between the presence of CNSV and leprosis symptoms, considering that no other extant virus was detected using diagnostic techniques, such as the PCR, RT-PCR and ELISA assays, as well as no additional RNA sequences were identified by deep-sequencing.

### 3.3. CNSV Particles Are Located in the Cytoplasm of Infected Cells

**Figure 5:**
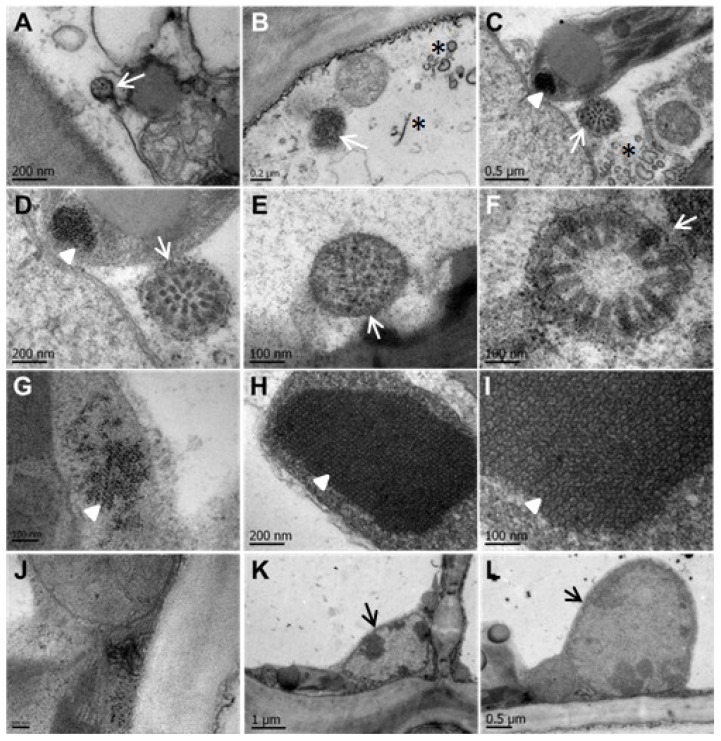
CNSV particles are located in the cytoplasm of infected cells. (**A**–**F**) Electron-dense viral particles are located in viroplasms within the cytoplasm of infected cells. Electron-dense structures with a membrane unit and average size of 120 to 475 nm (**A**–**F**) are indicated with arrows. A cross-section of a symmetric viroplasm, surrounded by host membrane, containing 16 rod-shaped viral structures, with a radial arrangement, is shown (**F**). Unassembled structures (**B**,**C**) are either linear or semicircular electron-dense filaments with an average length of 248 nm, indicated with asterisks. Electron-dense networks are present in the chloroplast of infected leaves (**C**,**D**,**G**–**I**; indicated with darts). (**J**) Membrane-associated viral particles in close proximity to the cell boundary. The nuclei of infected cells (**K**,**L**) without apparent viral structures. The bar size is indicated in each micrograph.

Considering CNSV belongs to a clade with similarity to cyto- and nucleo-rhabdovirus, it was necessary to determine the subcellular localization of CNSV in infected tissue. An analysis using transmission electron microscopy was carried out to determine the presence of virions in symptomatic sour orange (*Citrus* × *aurantium*) samples showing leprosis symptoms. As a control, the leaves of asymptomatic plants were also processed for image comparison. Rounded, membranous, electron-dense structures were identified in the cytoplasm with an average size of 120 to 475 nm (Figure 5A–F). The structure with the largest diameter (Figure 5F) shows a cross-section of a symmetric viroplasm, surrounded by a host membrane, containing 16 rod-shaped viral structures, with a radial arrangement. These structures suggest that the viroplasm is a sphere containing rod-shaped virion particles with an average size of 67 × 27 nm. Unassembled structures are either linear or semicircular electron-dense filaments with an average length of 248 nm. The theoretical length of CNSV RNA components (6018 and 6495 bases) is circa 200 nm; the observed filaments of 248 nm may correspond to the viral genomes likely associated with N nucleoprotein. Interestingly, an electron-dense network was present in the chloroplast of infected leaves (Figure 5C,D,G–I); 20 × 18-nm crystals showed a geometrical arrangement; similar inclusion bodies are formed in plant cells infected with several other viruses, but not in chloroplasts [51,52]. However, similar electron-dense bodies have been detected in the chloroplasts of plants infected with the *Bamboo mosaic virus* [53]. Such protein arrangements, as well as vesicles (Figure 5C) in the chloroplast were present only in infected cells and absent in asymptomatic samples. This suggests that this protein crystal network is of a viral nature or at least induced by viral infection [54,55].

**Figure 6:**
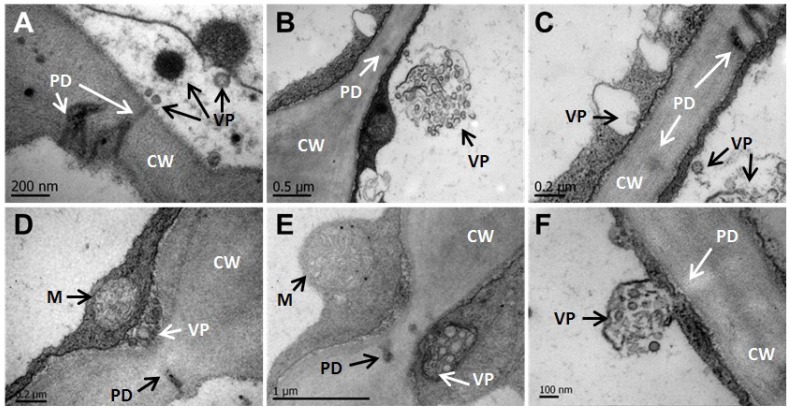
CNSV particles are associated with cell-to-cell interconnecting plasmodesmata. (**A**–**F**) Viroplasms (VP) and unassembled virions localize to secondary plasmodesmata (PD). Specialized membranous sacs show virus assembly-disassembly (**C**). Thinner cell walls (CW) with PD and viral particles (VP) are shown in (**D**,**E**). A disassembled viroplasm appears in close association with PD (**F**). M, mitochondria, CW, cell wall. The bar size is indicated in each micrograph.

Regarding the presence of viral particles in the nucleus, no evidence of electron-lucent viroplasms were observed, characteristic of Nucleorhabdovirus; indeed, the cytopathic effects of Nucleorhabdovirus, *i.e.*, large amounts of granular or fibrous viroplasms of various sizes occupying most of the nucleus and that are frequently observed near the nucleolus [50], were not observed in the analyzed tissue; instead, rare, small translucent areas were detected in the nuclei (Figure 6K,L). However, the presence of membranous material covering the viroplasm and its “spoke wheel” structure suggest that virus replication and virion formation could occur in the cytoplasm, possibly in the endoplasmic reticulum and in the outer nuclear envelope, in a manner reminiscent of Rhabdoviruses [17]. No budding of viral particles from nuclei was observed in the analyzed samples, supporting the notion that CNSV accumulation and replication do not occur in the nucleus, although it cannot be discarded that some stages of replication and nucleocapsid assembly do occur in the nucleus.

### 3.4. CNSV Particles Associate with Unmodified Plasmodesmata

TEM analysis showed numerous bacilliform virions and viroplasms associated with plasmodesmata (PD). Figure 6A shows two secondary PD associated with an electron-dense viroplasm; indeed, unassembled virions appearing as electron-dense filaments with an average length of 248 nm are in the close boundary of a PD, while a smaller and possibly immature viroplasm appears in the endoplasmic reticulum (ER) appressed onto the boundary of the neighboring cell (Figure 6B). In a magnified image, the transport of potential virion-associated macromolecules to the neighboring cell is suggested in (Figure 6C,F). On a speculative note, enveloped structures (probably virions) appear associated with the ER (Figure 6C–F). Interestingly, the thickness of the cell wall in the trafficking area (Figure 6D,E) is thinner than in other regions of the cell wall.

### 3.5. CNSV Elicits the Accumulation of Multiple Host Transcripts

Using mapped RNA-seq reads for both conditions, a total of 134 transcripts were found to differentially accumulate in symptomatic and asymptomatic leaf samples. One hundred eighteen of the corresponding transcripts displayed increased levels, while 16 were downregulated. Gene Ontology (GO) assignments were used to classify the functions of the up- and down-regulated genes. These fell into 12 categories of Level 2 GO terms (Tables S3 and S4; Figure S4), involved in the response to both biotic and abiotic stress, cell death, reproduction, development, metabolism, regulation, growth, signaling, localization and cellular organization and processes. Most of the genes with increased expression levels are involved in stress and defense responses, such as those encoding pathogenesis-related proteins 1 and 3, Mlo-like proteins, serine protease inhibitors, phenyl alanine ammonia lyase (PAL), BAHD acyl transferase-like proteins and chalcone synthase (CHS) [56,57]. Indeed, PAL and CHS are the central nodes for the synthesis of secondary metabolites, among them antimicrobial compounds [58,59]. Interestingly, some of the induced genes are involved in the defense response against mechanical damage and herbivory, such as a serine protease inhibitor (log2 fold-change = 4.8) and germin-like protein (log2 fold-change = 11.3). Germin-like proteins have been implicated in development and the defense response in various plant species [60]. The identity of other genes suggests a role in the response to viral pathogenesis, as in the case of a cysteine-rich receptor-like protein kinase (log2 fold-change = 6.0). Of note, several induced genes encode proteins involved in resistance against phytopathogenic fungi, such as *Cladosporium fulvum* (the Avr9 cf-9 rapidly elicited protein; log2 fold-change = 3.6) [61], which points to a non-specific response to viral infection. It must be mentioned that the control corresponds to an asymptomatic plant, and while extremely low viral levels were detected through HTS, it could still have elicited similar responses.

A graphic representation of the differentially expressed genes described above is shown in Figure S4, in which genes are classified according to cellular functions, metabolic processes, secondary metabolites and responses to biotic stress.

The deep sequencing of symptomatic and asymptomatic tissues revealed the unexpected presence of a phage with high similarity to ϕx174. Interestingly, there were approximately four-times more reads for this phage than for the total found for CNSV, although these were obtained in both symptomatic and asymptomatic leaves. It is likely that these RNAs correspond to an environmental sample from the phylloplane [62]. BLAST analysis of all the other assembled transcripts, in contrast, correspond to *Citrus* genes, disregarding that these may also be contaminants from the phylloplane.

**Figure 7:**
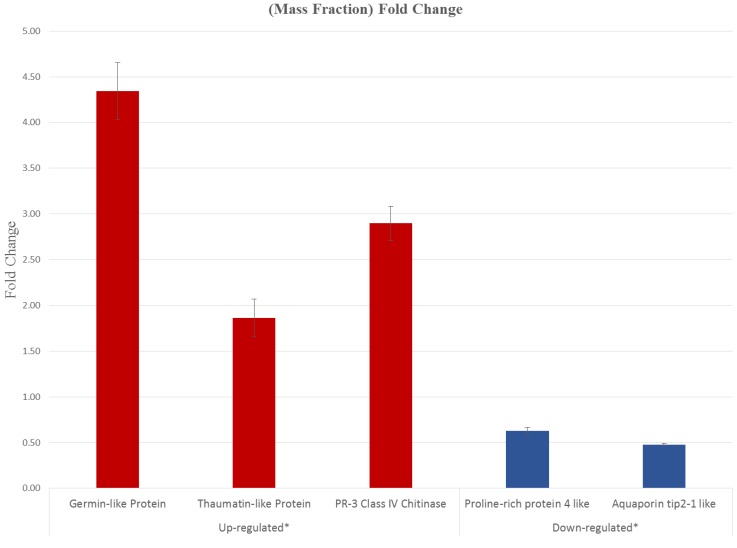
Validation of five differentially expressed genes through qRT-PCR. The fold change is statistically significant. Red, upregulated genes; blue, downregulated genes, according to RNA-seq analyzed data. The standard deviation is indicated with bars.

Considering the high log2 cutoff, few downregulated genes were identified. These include a heavy metal transport detoxifying protein, aquaporin TIP2, and a number of genes associated with primary metabolism. An alternate validation of five differentially expressed genes was performed by quantitative real time RT-PCR (Figure 7). The fold change was statistically significant using the F distribution (*p* = 0.05), thus confirming the differential expression of genes in virus-infected Citrus plants (Figure S4 and Tables S3 and S4).

Considering the differential expression through bioinformatic analyses, the genes annotated as germin-like protein subfamily 1 member 14, thaumatin-like protein, PR-3 class IV chitinase (Table S3) were validated through qRT-PCR (Figure 7), indicating that these genes (associated principally with the plant response against pathogens) are overexpressed throughout infected samples. Two downregulated genes encoding for the proline-rich protein 4-like and aquaporin tip-2-like identified in the RNA seq analyses were also confirmed to reduce their expression in infected tissue (Figure 7 and Table S4).

## 4. Discussion

Several diseases threaten the citrus industry, among them huanglongbing, citrus tristeza and citrus leprosis; the latter may be caused by different viruses, although eliciting common symptoms. Among the causal agents, HGSV [9] and, more recently, CiLV-C [7,11] and CiLV-N [10] have been already described. The etiological agent of nuclear leprosis, so-called because of the appearance of electron-lucent bodies in the nucleus, has been considered to be a species related to OFV [63].

### 4.1. CNSV Belongs to the New Proposed *Dichorhavirus* Genus

In this work, using deep sequencing of RNA from citrus plants showing leprosis symptoms, we identified a Dichorhavirus associated with leprosis symptoms, showing a high similarity to OFV, which was provisionally termed Citrus Necrotic Spot Virus (CNSV). The phylogeny of these viruses indicate that these fall within the same clade, though outside of the Nucleorhabdovirus and Cytorhabdovirus. Along with OFV and CiLV-N, they represent a unique clade of negative ssRNA virus, whose genome is bipartite. Since the global homology between CNSV and OFV/CiLV-N is close to 90%, these viruses can be considered strains or closely related species, although all belong to the dichorhavirus branch [6]. This clade is also very similar to a recently sequenced virus, Coffee ringspot virus (CRSV).

Rhabdoviruses are a diverse group of viruses infecting both plants and animals; aside from the negative polarity of their ssRNA genome, they all share the lipidic membranous envelope surrounding its capsid. The nuclei of infected cells harbor electron-lucent regions, as has been observed with OFV and nucleorhabdoviruses (OFV) [63,64]. The electron microscopy data presented here suggests that CNSV does not form viroplasms in the nuclei of infected cells (Figure 6K, L). Additionally, CNSV belongs to an unusual clade that includes OFV, which has been extensively described [6,63,64]. Bullet-shaped virus particles, a hallmark of rhabdovirus, were not observed; instead, long filaments that could consist of RNA and viral and/or host proteins were detected in large spoke-wheels structures, similar to those observed in OFV-infected *Odontoglossum* [50,55,64]. Interestingly, these structures reside in the cytoplasm, and no nucleus-budding viral structures could be identified in the analyzed nuclei; however, no CiLV-N ultrastructure is available for comparison. Furthermore, large electron-dense networks were observed within chloroplasts of CNSV-infected cells. It is possible that these large aggregates (perhaps consisting of viral and host proteins) may interfere with chloroplast function, causing some of the macroscopic symptoms, such as chlorosis. The close association of vesicles, possibly harboring viral RNA and filamentous particles, with plasmodesmata is suggestive of the mechanism for CNSV cell-to-cell transport, *i.e.*, through unmodified plasmodesmata. Indeed, no evidence was found for tubular structures formed during the intercellular transport of certain plant viruses. Experiments with protoplasts using labeled viral proteins, in particular the movement protein, which is probably involved in cell-to-cell transport, will help to clarify this question.

### 4.2. CNSV Induces a Characteristic Set of Genes during Infection

Mites of the genus, *Brevipalpus*, widely distributed in the studied areas, transmit leprosis disease in citrus. In general, viruses transmitted by these vectors do not move systemically in an extensive manner. Rather, they move cell-to cell, thriving in sectors of leaves and fruits, and it is likely that they do not reach the vasculature. Since most signals activating the defense response against pathogens are transported through the vascular tissue [65], this could result in a slower activation of a systemic response. Viral infection was found to induce strongly a germin-like protein mRNA. These proteins may have a role in defense to biotic and abiotic stress [58] and development [66], and the overexpression of a member of this large family in tobacco increases resistance to Geminivirus infection [67]. Interestingly, a beta glucanase encoding transcript was highly accumulated; this is a non-cell-autonomous pathway protein (NCAPP), described as increasing the gating capacity of plasmodesmata and, thus, facilitating viral movement [68]. While differentially regulated genes fell in several GO categories, most (54%) are classified within the stress response and defense categories. Indeed, tissue necrosis, if limited, is a plant response that results in pathogen limitation. Proteases and cell wall remodeling activities, among others, account for the slow progression of the cell-to-cell viral movement. Transcripts for proteins involved in cell signaling and innate plant immune response accumulate to considerably high levels; all of these data strongly suggest the notion of a coordinate plant defense against the pathogen. Secondary metabolism could also play an important role in response to viral infection, given that RNAs for two key enzymes, PAL and CHS, accumulate to high levels. PAL is the key enzyme in phenylpropanoid biosynthesis, and CHS is involved in the synthesis of flavonoids, among several other metabolites. However, it is not clear whether these responses have an essential role in slowing virus replication and/or spread. It must be mentioned that the relatively low number of differentially expressed genes is due to the high cutoff value set (a log2 fold value), and that asymptomatic plants are subliminally infected. Furthermore, several reads that did not match the *C. × clementina* or *C. sinensis* genomes could correspond to genes specific to *C.* × *aurantium* and, in particular, to untranslated regions or even small RNAs. The observed set of stress- and defense-related genes altering their expression profiles in response to viral infection are in agreement with those identified in general plant responses to viral infections [69,70].

Ongoing epidemiological studies will provide valuable information regarding the extent of Citrus Necrotic Spot Virus infections in Mexico, one of the main citrus producers in the world.

## Supplementary Files

Supplementary File 1 Supplementary Materials (PDF, 1670 KB)
