# Supplementary material for: Characterization of a Proposed Dichorhavirus Associated with the Citrus Leprosis Disease and Analysis of the Host Response
Source: Viruses. 2014 Jul 7;6(7):2602–22. doi: 10.3390/v6072602 (PMC4113785; doi:10.3390/v6072602)
Supplement: Supplementary File 1 — Supplementary Materials (PDF, 1670 KB) [file viruses-06-02602-s001.pdf]

Supplementary Materials

Characterization of a Proposed *Dichorhavirus* Associated with the Citrus Leprosis Disease and Analysis of the Host Response

Figure S1. CiLV-C and HGSV are not present in citrus samples displaying leprosis symptoms. (**A**) Agarose gel showing no amplified cDNA when total RNA was used as a template sour orange tissue when primers directed against the CP of CiLV-C (**upper panel**) and HGSV (**middle panel**). (**Lower panel**) Amplification of the internal control, COX, from the *Citrus* species. (**B**) Primers employed in the detection of infected tissue.

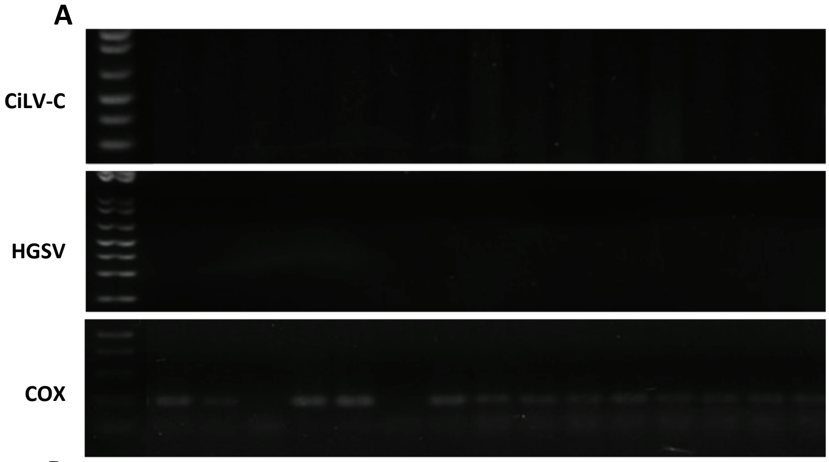

B

CILV-C MP 5'	TCGTTTGGTTGGGTTCATAAACCGTA
CILV-C MP 3'	TATACCAAGCCGCCTGTGAACTTCAG
HGSV 5'	GCGACGGGAAAAATACTGAAGCATAG
HGSV 3'	GTGCAGCATCCAAAATCTCCACCTTC
CITRUS COX 5'	GAGGCATTTTTGGATCACTTTT
CITRUS COX 3'	GTTCTGTTAGGTTCTTAGTAGC

Figure S2. Deep-sequence read coverage of the RNA 1 and RNA2 of the CNSV genome. These are represented as green and red lines under the assembled viral genome. Green lines correspond to sense reads relative to the OFV genome and red lines to antisense reads.

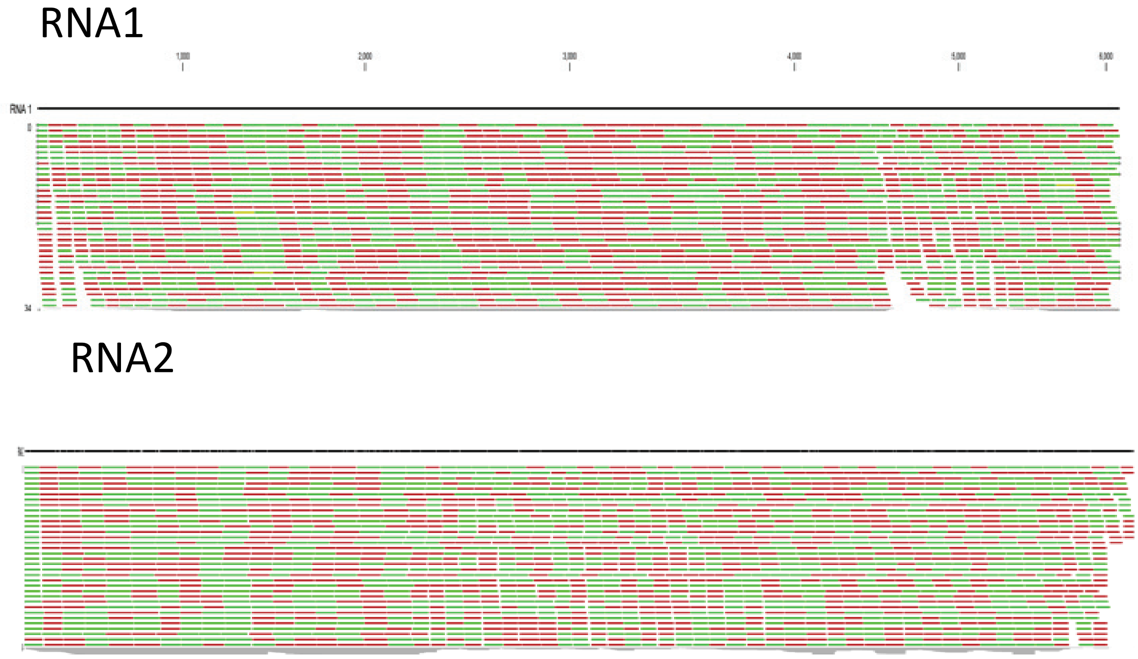

Cruz-Jaramillo *et al.*, 2014.

Figure S3.

Comparison of RNA1 .

```
                Leader
CNSV      AAGCAGTGGTATCAACGCAGAGTACATGGGGGTCTTCTAGTGCAAGTGGCAGAAATGACC 60
CILV-N    ACACAG-GATAACC-CGC--------TATTGGTCTTCTAGTGCAAGTGGCAGAAATGACC 50
OFV       ACACAG-GATAACC-CGC--------TATTGGTCTTCTAGTGCAAGTGGCAGAAATGACC 50
          *  *** * ** *  ***        *   ******************************

CNSV      GCTTAAACAGATCCCCCTAAGTTTGGATGCTGAGCTAGCGGTAGTGTATTGGTGAGTC 120
CILV-N    GCTTAAACAGATCCCC-TAAGTTTGGATGCTGAGCTAGCGGTAGTGTATTGGTGAGTC 109
OFV       GCTTATACAGATCCCC-TAAGTTTGGATGCTGAGCTAGCGGTAGTGTATTGGTGAGTC 109
          ***** ********** *******************************************

CNSV      CTTCTCCCTTAGCTACGGTCACAGCCCTGGGTACATGATGTAAACAAAAAAGCAGTGGTA 180
CILV-N    CTTCTCCCTTAGCTACGGTCACAGCCCTGAGTACATGATGTAAACAAAA----------- 158
OFV       CTTCTCCCTTAGCTACGGTCCCAGCCCTAAATACATGATGTAAACAAAA----------- 158
          ******************** *******   ******************

CNSV      TCAACGCAGAGTACATGGGGAAAGTGGATAATAACTACACTGATTGGACAAAATTGTACT 240
CILV-N    -CAAC----------------AAGTGGATAATAACTACATTGATTGGACAAAATTGTACT 201
OFV       -CAAC----------------AAGTGGATAAAAATTGCATTGATTGAACAAAATTGTACT 201
           ****                **********  ** * ** ****** *************
                                          ORF1: N
CNSV      AATATATCTCTCTCTACGTAGGAAACATGGCTAATCCAAGTGAGATCGATTACATGACTC 300
CILV-N    AATATATCTCTCTCTACGTAGGAAACATGGCTAACCCAAGTGAGATCGATTACATGACTC 261
OFV       AATATTTCTCTCTCTACGTAGGAAACATGGCTAACCCAAGTGAGATTGATTACATGACTC 261
          ***** **************************** *********** *************

CNSV      CTCTCTCTGCATACGAGGGGGTTCCCGCCGAATATCAGGAAGCAACAAGCTCCCCAACGC 360
CILV-N    CTCTCTCTGCATACGAGGGGGTTCCCGCCGAATATCAGGAAGCAACAAGCTCCCCAACGC 321
OFV       CCCTCTCTGCATACGAGGGAGTTCCTGCTGAATACCAAGAAGCAACAAGCTCCCCAACAC 321
          * ***************** ***** ** ***** ** ******************** *

CNSV      CAAAGGAATACACCAGAGATGCGGCCAAAGCAATCCCAATATGCACTCTTCCTGCCCCGC 420
CILV-N    CGAAGGAATATACCAGAGATGCAGCCAAAGCAATCCCAATATGCATTCTTCCTGCCCCAC 381
OFV       CGAAGGAATACACCAGAGATGCAGCCAAAGCAATCCCAATATGCATCCTCCCTGCCCCGC 381
          * ******** *********** **********************  ** ******** *

CNSV      CAGGTAATGAGGTCGAGGTGGCTGAAGCCTTCAGGGAGGCCACACAGGGCACCGAGACAG 480
CILV-N    CAGGTAATGAGGTCGAGGTGGCTGAAGCCTTCAGGGAGGCAACACAGGGCACCGAGACAG 441
OFV       CAGGCAATGAGGTTGAGGTGGCTGAAGCCTTCAGAGAGGCAACACAGGGCACTGAAACGG 441
          **** ******** ******************** ***** *********** ** ** *

CNSV      TCCTGTCAAAGTTGCAGTTGGCACAAATAATGTCTCTGGGGTTCATGGTCCAGATGTCGG 540
CILV-N    TCCTGACAAAGTTGCAGTTGGCACAAATAATGTCTCTGGGGTTCATGGTCCAGATGTCGG 501
OFV       TCCTGTCAAAGTTGCAACTGGCACAAATAATGTCTCTGGGATTTATGATCCAGATGTCGG 501
          ***** **********  ********************** ** *** ************

CNSV      GGGATCCTGAGGCATTAATGCAGGGAGTGGCAGAGGGTTCGTTCCTGGAGACCATGACCA 600
CILV-N    GGGATCCTGCGGCATTAATGCAGGGAGTGGCAGAAGGTTCATTCCTGGAGACCATGACCA 561
OFV       GAGATCCTGAGGCATTGATGCAGGGAGTGGCAGAGGGGTCATTCCTGGAGACCATGACCA 561
          * ******* ****** ***************** ** ** *******************

CNSV      TGCCGGACATCAAGACAAGGCAGGACAGCCGGATCACAGCAGCCATTGCAGCCATGGCAT 660
CILV-N    TGCCGGACATCAAGACAAGGCAGGACAGCCGGATCACAGCAGCCATTGCAGCCATGGCAT 621
OFV       TGCCGGACATTAAGACGAGGCAAGACAGCAGGATCACAGCAGCCATAGCAGCCATGGCAT 621
          ********** ***** ***** ****** **************** *************

CNSV      TGGACCCAACGGAGGAGGGGCCAGATGACCCGGAAGATAGATCTCAGCAGGCTGATGCAG 720
CILV-N    TGGACCCAACAGAGGAGGGGCCAGATGACCTGGAAGACAGATCTCAGCAGGCTGAGGCAG 681
OFV       TGGACCCAACAGAGGAGGGGCCAGATGACCTGGAGGACAGATCTCAGCAGGCAGATGCGG 681
          ********** ******************* *** ** ************** ** ** *

CNSV      AGAGTGCACTGTCCAAGGCTAGGGCAATGGTATACATATGCTTAAGCCTTATGAGGCTAG 780
CILV-N    AGAATGCACTGTCCAAGGCTAGGGCAATGGTATACATATGCTTAAGCCTTATGAGGCTAG 741
OFV       AGAGTGCGATGTCCAAGGCCAGAGCAATGGTGTACATATGCTTAAGCCTCATGAGACTGG 741
          *** ***  ********** ** ******** ***************** ***** ** *
```

```
CNSV        CTGTGAAGCCTGCTGAGTCATTCATGAAGGGTGTTCATCAGATTAAACAGGCATATTCAG 840
CILV-N      CTGTGAAGCCTGCTGAGTCATTCATGAAGGGTGTTCATCAGATCAAACAGGCATATTCGG 801
OFV         CTGTGAAGCCGGCTGAGTCATTTATGAAGGGTGTTCATCAGATCAAACAGGCATATTCAG 801
            ********** *********** ******************** ************** *

CNSV        TCCTGGTGGGAGAGCACTCTGAGTTCCTGTTCAATTATTCATACTCAGAAGGAATGTGCA 900
CILV-N      TCCTGGTGGGAGAGCACTCTGAGTTCCTGTTCAATTATTCATACTCAGAAGGAATGTGCA 861
OFV         TCCTGGTAGGAGAGCATTCTGAATTCCTGTTCAATTACTCCTATTCAGAAGGAATGTGCA 861
            ******* ******** ***** ************** ** ** ****************

CNSV        GAAACATAGCCGACATGTTCAACCAATGCGATGATCTCAAGGCAACTCTTTGTCATCATT 960
CILV-N      GAAACATAGCCGACATGTTCAACCAATGTGATGATCTCAAGGCAACTCTTTGTCATCATT 921
OFV         GGAATATAGCCGACATGTTCAACCAATGTGATGATCTCAAAGCAACTCTTTGCCATCACT 921
            * ** *********************** *********** *********** ***** *

CNSV        GTGCCATAGCAGATGAAACGCACCACACCAATAGGAAGAGGCACGGGCTTCTCAGATTCC 1020
CILV-N      GTGCCATAGCAGATGAAACGCACCACACCAATAGGAAGAGGCACGGGCTTCTCAGATTCC 981
OFV         GCGCCATAGCAGATGAGACGCACCACACCAATAGGAAGAGGCACGGACTTCTCAGATTCC 981
            * ************** ***************************** *************

CNSV        TGATCCTTCAGCATGTAGATCTCACAGGGATGATCCCATATGGGATGTACATTGACATGA 1080
CILV-N      TGATCCTTCAGCATGTAGATCTCACAGGGATGATCCCATATGGGATGTACATTGACATGC 1041
OFV         TAATCCTTCAACATGTGGATCTTACGGGGATGATCCCATATGGAATGTACATTGACATGC 1041
            * ******** ***** ***** ** ***************** *************** 

CNSV        GAAGGCACTTCACCCTCCTGACACCCGGCCAGTTGCTGACATGGCTCCATGACAACCAGG 1140
CILV-N      GAAGGCACTTCACCCTCCTAACACCCGGCCAGTTGCTGACATGGCTCCATGACAACCAGG 1101
OFV         GAAGGTACTTCACCCTCTTAACACCCGGCCAGTTGCTAACATGGCTTCATGACAACCAGG 1101
            ***** *********** * ***************** ******** *************

CNSV        TATCCAGGCCTCTCAGTGTCATTGCGGACATAAACACCAGGTATGATGTTTCAAACGGAT 1200
CILV-N      TATCCAGGCCTCTCAGTGTCATTGCGGACATCAACACCAGGTATGATGTTTCAAACGGAT 1161
OFV         TATCTAGGCCTCTCAGTGTGATTGCGGACATAAACACCAGGTATGATGTTTCTAACGGGT 1161
            **** ************** *********** ******************** ***** *

CNSV        CAGACCGGTTCTGGAGGTACTCAAGGGGGCTTGATCCAGGATTCTTCATAGCATTACAAC 1260
CILV-N      CAGACCGGTTCTGGAGATACTCAAGGGGGCTTGATCCAGGATTCTTCATAGCCTTACAAC 1221
OFV         CAGATCGGTTCTGGAGGTACTCAAGGGGTCTAGACCCAGGATTCTTTATAGCTTTACAAC 1221
            **** *********** *********** ** ** *********** ***** *******

CNSV        AATCAAAATGTGTCACCTTGATAGCCAGAATGGCACACATACTGGTGAAGGGAGGAGCCG 1320
CILV-N      AATCAAAATGTGTCACCCTGATAGCCAGAATGGCACATATACTGGTGAAGGGAGGAGCCG 1281
OFV         AGTCAAAATGTGTCACTCTGATAGCCAGGATGGCACATATACTGGTGAAGGGAGGAGCCG 1281
            * **************  ********** ******** **********************

CNSV        TTGCTGTCAATGAATACTCCGACCCCCGAAAGGCAAAGTCACTGGAGAACAAGCCTGGGC 1380
CILV-N      TTGCTGTCAATGAGTACTCCGACCCCCGAAAAGCAAAGTCACTGGAGAACAAGCCTGGGC 1341
OFV         TCGCTGTCAATGAATACTCCGATCCTCGAAAGGCAAAGTCATTGGAGAACAAGCCTGGGC 1341
            * *********** ******** ** ***** ********* ******************

CNSV        TGGCAGCGGAGGCTGACAAGTTTGCCACAGAGTTTGTGGAAGCATACAACGGCCTATCAG 1440
CILV-N      TGGCAGCAGAGGCTGACAAGTTTGCCACAGAGTTTGTGGAAGCATACAACGGCCTATCAG 1401
OFV         TGGCAGCGGAGGCAGACAAGTTCGCCACTGAGTTCGTGGAGGCATACAACGGCCTATCAG 1401
            ******* ***** ******** ***** ***** ***** *******************

CNSV        GGTCAAGTGCTAATGCCGGCCCTGTATCCCGTAAACTCTACAACCAAGGCAGAGGTATTC 1500
CILV-N      GGTCAAGTGCTAATGCCGGCCCTGTGTCCCGCAAACTCTACAACCAAGGCAGAGGTATTC 1461
OFV         GATCAAGTGCAAACGCCGGCCCTGTGTCCCGCAAACTCTACAACCAGGGCAGAGGCATCC 1461
            * ******** ** *********** ***** ************** ******** ** *

CNSV        CTACCCGCAGAGGCCTATTCACACCTCCCTCCGCCAGACCCGCACCTGTTGTGAATGTGC 1560
CILV-N      CTACCCGCAGAGGCCTATTCACACCTCCCTCCGCCAGACCCGCACCTGTTGTGAATGTGC 1521
OFV         CGACCCGCAGGGGCCTGTTCACACCCCCTTCCGCCAGACCTGCGCCCGTTGTGAATGTAC 1521
            * ******** ***** ******** ** *********** ** ** *********** *


CNSV        ATATCCCCGCCGCCTCGTCCTCTTTAGCAGGAGCCCTGGATGCTATGAACAGTGAATGAC 1620
CILV-N      ACGTCCCAGCCGCCTCGTCCTCTTTGGCAGGAGCCCTAGATGCTATGAACAGCGAATGAC 1581
OFV         ACGTCCCTGCCGCCTCGTCCTCTCTGACAGGGGCCCTGGATGCCATGAACAGTGACTGAC 1581
```

```
                  *  **** *************** *  **** ***** ***** ******** ** ****
                  IGR1
CNSV              AACCATCCTCTCATGGCAAACTTCCATCCCTACCATGACAATATGCTATATATATATGCG 1680
CILV-N            AACCATCCTCTCATGGCAAACTCCCATCCCTATCGTGACTACATGCTATATATGT----G 1637
OFV               AGCCACCTCTCCATGGAAGAC-CCCATCTCTATCATAACAATATGCTATATATGC----G 1636
                  * *** *    ***** * **  ***** *** * * ** * ***********      *

CNSV              TGCATTTAAATAAAAAAAAAACAGGAAAACTTATGCTTGTTCATCTCCTCACGCACACAC 1740
CILV-N            TGCATTTAAATAAAACAACAACAGGGAAACTTATGTTTGCTCATCTCCTCACGCACACAC 1697
OFV               TGCATTTAAATAAAACAACAACAGCGGAACTTGTGTTTGTTTATCACTTCAT----ATAT 1692
                  *************** ** *****     ***** ** *** * *** * ***     * *
                                                     ORF2: P
CNSV              ACTAACGGCCTCACATATATTCTCTTTCCAGGAAAATGTTCACCACCAAGGTAAATATGT 1800
CILV-N            ACTAACAGCCTCACATATATTCTCTTTCCAGGACAATGTTCACCACCAAGGTAAATATGT 1757
OFV               ATTAACAGCTTCACATATATTCTCATTCCAGGGAAATGTTCACCACCAAGGTAAATATGT 1752
                  * **** ** ************** *******  **************************

CNSV              ACCCAGAGGTCCCCAGCTCGTCTCAAGTGTCCGATGACATAGACAACGACACACAAGTAG 1860
CILV-N            ACCCAGAGGTCCCCAGCTCGTCTCAAGTGTCCGATGACATAGACAACGACACAAACATAG 1817
OFV               ACCCAGAGGTCCCCAGCTCATCCCAAGTGTCCGACGACATAGACAACGACACACACGTAG 1812
                  ******************* ** *********** ****************** *  ***

CNSV              ACGAGGTCGCAGCATTTGTGAGGAAATGGTCGGCTGCCGGACTATCTCCCCCTGTCACCC 1920
CILV-N            ACGAGGTCGCAGCATTTGTGAGGAAATGGTCGGCTGCCGGACTATCTCCCCCTGTCACCC 1877
OFV               ATGAGGTCGCAGCATTTGTGAGGAAATGGTCAGCTGCCGGACTATCTCCCCCGTCACCC 1872
                  * ***************************** ******************** *******

CNSV              TTGCAAAGAACCTTAGAGCATGGATAGCAAGCAACACCAACCCAGGGAACCCCTTGGTGC 1980
CILV-N            TTGCAAAGAACCTTAGAGCATGGATAGCAAGCAACACCAACCCAGGGAACCCCTTGGTGC 1937
OFV               TTGCAAAGAACCTCAGAGCATGGGTAGCGAGCAACACCAACCCAGGGAACCCCTTGGTGC 1932
                  ************* ********* **** *******************************

CNSV              TGGACGACAGGATGCTAAGTCTCACAACCATGATATGGAACACAGCAGCGGAGCATTATA 2040
CILV-N            TGGACGACAGGATGCTAAGTCTCACAACCATGATATGGAACACAGCAGCGGAGCATTATA 1997
OFV               TGGACGACAGGATGCTTAGTCTCACGACCATGATATGGAACACAGCAGCGGAGCATTATA 1992
                  **************** ******** **********************************

CNSV              CTATGATCGGCAAATCCCAGGTCAATCGCATGTCATCACTCATCGACCAGCTGGGGGAGA 2100
CILV-N            CCATGATCGGCAAATCCCAGGTCAATCGCATGTCATCACTCATTGACCAGCTGGGGGAGA 2057
OFV               CTATGATCGGTAAATCTCAGGTCAATCGTATGTCAGCGCTTATTGACCAGCTGGGGGAGA 2052
                  * ******** ***** *********** ****** * ** ** ****************

CNSV              TCTCCGGCCGCAGACCACAGCAGGGTCCCTCGCTCGATATGCCACCTCCCCCTCCTAAGA 2160
CILV-N            TCTCCGGCCGCAAGCCACAACAGGGTCCCTCGTTCGATATGCCACCTCCCCCTCCTAAGA 2117
OFV               TCTCCGGCCGAAAACCGCAGCAGGGTCCCTCATTCGACATGCCACCTCCCCCTCCTAAGA 2112
                  ********** *  ** ** ***********  **** **********************

CNSV              GGAAACACCCAGATTCACTAGACACTAACCCTATCTTAGGCTTAATAGGTCAGGACTGGG 2220
CILV-N            GGAAACACCCAGACTCTCTAGACACTAACCCTATTTTAGGCTTAATAGGTCAGGACTGGG 2177
OFV               GGAAACACCCGGATTCACTAGACACTAACCCTATCTTAGGCTTAATAGGTCAGGACTGGG 2172
                  ********** ** ** ***************** *************************

CNSV              ATGAAAAGAAGGACAAACAGTGGAGGGAGAAGCCAGCAGACAAGAAACATCTAGTGCTCA 2280
CILV-N            ACGAAAAGAAGGACAAACAGTGGAGGGAGAAGCCAGCAGACAAGAAACATCTAGTGCTCA 2237
OFV               ACGAGAAGAAGGACAAACAGTGGAGGGAGAAGCCAGCAGATAAGAAACATCTAGTGCTCA 2232
                  * ** *********************************** *******************

CNSV              ACTGGGTGTTACACGAGTACCTGGGGGTTCTAACAAAACCGGTAACAATCAAGTGGATAA 2340
CILV-N            ACTGGGTGTTACACGAGTACTTGGGAGTCCTAACAAAACCGGTGACAATCAAGTGGATAA 2297
OFV               ACTGGGTGTTACATGAATATTTGGGAGTCCTAACAAAACCGGTAACGATCAAGTGGATAA 2292
                  ************* ** **  **** ** ************** ** *************

CNSV              CAGACAATCCATCGTCCCTGGAGTTGGGAGCAGTTTCAGCATATGCCCTAAGACATCAGG 2400
CILV-N            CAGACAATCCATCGTCCCTGGAGTTGGGAGCAGTTTCAGCGTATGCCCTAAAACATCAGG 2357
OFV               CAGACAATCCGTCGTCTCTGGAGTTGGGAGCAGTTTCAGCATACGCCCTGAAACATCAGG 2352
                  ********** ***** *********************** ** ***** * ********

CNSV              CCAGCTTGTCCGACTGCGATAAAGAGGCCCTCAGAGCACTCGTGGTCCAGACAGTGAAGA 2460
CILV-N            CCAGCTTGTCCGACTGTGATAAAGAGGCCCTCAGAGCACTCGTGATCCAGACAGTGAGGA 2417
OFV               CCAGCTTGTCCGACTGTGACAAAGAGGCCCTCAGAGCACTTGTGGTTCAAACAGTGAAGA 2412
```

```
                    **************** ** ******************** *** * ** ******* **
                                                       IGR2
CNSV                ACACCCCCAAGAGGCCATGCCTGGACTAGGTGATATCCTATCAGCCCTATCATATATATG 2520
CILV-N              ACACCCCCAAGAGGCCATGCCTGGACTAGGTGATATCCTATCAGCCCTATCATATATATG 2477
OFV                 ACACCCCCAAAAGGCCATGCCTGGACTAGGTGATATTCTATCAGCTTTATCATATATATG 2472
                    ********** ************************* ********  *************

CNSV                TTTCTCATTGTCAGTCATGCATATGATAATAGAGATGCTCAACAGTGACTCACCAGACTC 2580
CILV-N              TTTCTCATTGTCAGTCATGCATATGATAATAGAGATGCTCAACAGCGACTCACCAGACTC 2537
OFV                 TTTCTCATTGTCTGTCATGCATATGATAATAGAAATGCTCAATAGTGACTCACCGGACTC 2532
                    ************ ******************** ******** ** ******** *****
                                                                ORF3:   MP
CNSV                CATAATATATGTACTAGGCATTATGCAGTACGTTGTTGTGATACATGCATTTAAATAACA 2640
CILV-N              CATAATATATGTACTAGGCATTATGCAGTACGTTGTTGTAATACATGCATTTAAATAAAA 2597
OFV                 CATAATATATGTACTAGGCATTATGCAGTACGTCGTTGTAATTCATGCATTTAAATAAAA 2592
                    ********************************* ***** ** *************** *

CNSV                CATCAACTAATGTCATTGACTGGTATCATATTTGCTATAATTATAATATATATACTTGTG 2700
CILV-N              CATCAACTCATGTCATTGACTGGTATCATATTTGTGATAATTATAATATATATACTTTTG 2657
OFV                 CATCAACTTATGTCATTAACTGTTATCATATTTTCTATAATCATAATATATATACTTGTT 2652
                    ******** ******** **** **********   ***** *************** *

CNSV                CTTGTACTGACCCTCCTGCAGGATCTAAGATGTCCACATTGCCAGTGTCCACTCTTCACA 2760
CILV-N              CTTATACTGACCCTCCTGCAGGATCTAAGATGTCCACATTGCCAGTGTCCACTCTTCACA 2717
OFV                 CTTGTGTCAACCATCCTGCAGGAGCTAAGATGTCCACACTGCCAGTGTCTACTCTTCACA 2712
                    *** *    *** ********** ************** ********** **********

CNSV                GGTCCAACGCTAGGGACGAGGTCCGAGAGACGGCAGGCCGTAGGATGGCCCACGGGTTCG 2820
CILV-N              GGTCCAACGCTAGGGACGAGGTCCGAGAGACGGCAGGCCGTAGGATGGCCCACGGGTCCG 2777
OFV                 GGTCCAACGCTAGGGACGAGGTCAGGGAGACGGCAAGTCGTAGGATGGCCCACGGGTCCG 2772
                    *********************** * ********* * ******************* **

CNSV                ACAGCAATGTGACAGACTTCAACAAGAATCTGCAAACTGCTGAGGCTTGGCAGAAACGTC 2880
CILV-N              ACAGCAATGTGACAGACTTCAACAAGAATCTGCAAACTGCTGAGGCTTGGCAGAAACGTC 2837
OFV                 ACAGCAATGTGACGGACTTCAACAAAAATCTGCAAACTGCTGAGGCTTGGCAGAAACGCC 2832
                    ************* *********** ******************************** *

CNSV                TAATACACAAGACAACCATTAAGGTGACCGGAACAGAGGGTGAGGGAAGGATCACCATGG 2940
CILV-N              TAATACACAAGACAACCATTAAGGTGACTGGGTCGGAGGGTGAGGGAAGGATCACCATGG 2897
OFV                 TAATACACAAAACGTCCATCAAGGTGACTGGGTCGGAAGGTGAGGGGAGGATCACCATGG 2892
                    ********** **  **** ******** **  * ** ******** *************

CNSV                TGAGGAAGCCCATATTGTTTGACATCATGAACAGCCTGAGCAGCATACTTCCCACATCAA 3000
CILV-N              TGAGGAAGCCCATATTGTTTGACATCATGAACAGCCTGAACAGCATACTTCCCACATCAA 2957
OFV                 TGAGAAAGCCCATACTGTTTGACATCATGAACAGTCTAAACAGCATACTTCCCACATCGA 2952
                    **** ********* ******************* ** * ****************** *

CNSV                CAAAACCTACATGGATCATGGGGATGGCCATTAAATGGGTCCCAACATGTGACCTATCCA 3060
CILV-N              CGAAACCTACATGGATCATGGGGATGGCCATTAAATGGGTCCCAACATGTGACCTATCCA 3017
OFV                 CAAAGCCAACATGGATCATGGGGATGGCCATCAAGTGGGTTCCGACATGTGACCTGTCCA 3012
                    * ** ** *********************** ** ***** ** *********** ****

CNSV                CCACAGGGACCATCAAAGTATCGATTCAGAATAAGGCTGTCAATAATCCTGTTCTAAGGG 3120
CILV-N              CCACAGGGACCATCAAAGTGTCGATTCAGAATAAGGCTGTCAGTAACCCTGTTCTAAGGG 3077
OFV                 CAACAGGGACCATCAAGGTATCAATCCAGAATAAGGCTGTCAACAATCCTGTTTTAAGGG 3072
                    * ************** ** ** ** ****************  ** ****** ******

CNSV                ATCATACTGTCGTCTCCATGACCCAAAGAGCTACAACCCCATTTGAAGTTCAATACACAT 3180
CILV-N              ATCATACTGTTGTCTCCATGACCCAAAGAGTTACAACCCCATTCGAAGTCCAATACACAT 3137
OFV                 ATCACACTGTTGTCTCCATGACCCAAAGAGTCACAACCCCATTTGAGGTCCAATACACAT 3132
                    **** ***** *******************  *********** ** ** **********

CNSV                CATCCTCCAAGCTCGCCAATAGAACAGGCACAAGGGGGAATCCCTGGATGTATACATACT 3240
CILV-N              CATCCTCCAAGCTCGCCAATAGAACAGGCACAAGGGGGAATCCCTGGATGTATACATACT 3197
OFV                 CATCCTCCAAGCTAGCCAACCGGACAGGCACGAGGGGGAATCCCTGGATGTATACATATT 3192
                    ************* *****  * ******** ************************** *


CNSV                GCATAGAGGGAATGGATGACGCTCCGATAGACATGGAGGTGGGGGATATTGTTGTTATGC 3300
CILV-N              GCATAGAGGGAATGAATGACGCTCCGATAGACATGGAGGTGGGGGATATTGTTGTCATGC 3257
OFV                 GCATAGAGGGAATGGATGACGCCCCGATAGACATGGAGGTGGGGGATATTGTTGTTATGC 3252
```

```
                 ************** ******* ********************************* ****

CNSV             CCATGATTAAGTCTGATGAAACTAATACACAGTGGTATGAGGGCGTGAAGTGTAATGTGT 3360
CILV-N           CCATGATCAAGTCTGATGACACTAATACACAGTGGTATGAGGGCGTGAAGTGTAATGTGT 3317
OFV              CTATGATTAGGTCCGATGACACCACTACACAGTGGTATGAGGGTGTGAAGTGCAATGTGT 3312
                 * ***** * *** ***** ** * ****************** ******** *******

CNSV             ATGGGGGTTATTTTCCTCTGAACATACCTGTGGTCACCTACTGTGCACCCGGACCTAGGT 3420
CILV-N           ATGGGGGTTATTTCCCTCTGAACATACCTGTGGTCACCTACTGTGCACCCGGACCTAGGT 3377
OFV              ATGGAGGTTACTTCCCTCTGAATATACCGGTGGTCACCTACTGTGCACCCGGACCTAGGT 3372
                 **** ***** ** ******** ***** ******************************

CNSV             TCAAGACCAATATGAATGAGATAAGGTCAAACATAGAAATGCTCAGGAGGTATCTCAACG 3480
CILV-N           TCAAGACCAATATGAATGAGATAAGGTCAAACATAGAGATGCTCAGGAGGTACCTCAACG 3437
OFV              TCAAGACCAATATGAATGAGATAAAATCAAACATAGAGATGCTCAGGAGGTATCTCAACG 3432
                 ************************  *********** ************** *******

CNSV             TCCAGGGGTTCACTGATATAGATGAGGATCTGGTCTTCAAAATGATACAGTGCTGCGACG 3540
CILV-N           TCCAGGGGTTCACTGATGTGGATGAGGATTTGGTCTTCAAAATGATACAGTGCTGCGACG 3497
OFV              TCCAGGGATTTACCGATATAGATGAAGATCTGGTCTTCAAAATGATACAGTGTTGCGACG 3492
                 ******* ** ** *** * ***** *** ********************** *******

CNSV             GAGAGGCTGCAGGGTCCATAATGAAAGGGATAAGATCCTCCGTGTGGGCCCCTTTAGCTA 3600
CILV-N           GAGAGACTGCAGGGTCCATAATGAAAGGGATAAGATCCTCCGTGTGGGCCCCTTTAGCTA 3557
OFV              GGGAGACTGCAGGATCTATAATGAAGGGGATAAGATCCTCAGTGTGGGCCCCTTTGGCCA 3552
                 * *** ******* ** ******** ************** ************** ** *

CNSV             AGACTGACAGGGATTATGTTGTTATTAAGGAGATGCTCCACGACTGTCGTGTGGGCAGAA 3660
CILV-N           AGACTGACAAGGATTATGTTGTCATTAAGGAGATGCTTCATGACTGTCGTGTGGGCAGAA 3617
OFV              AGACTGACAGGGATTATGTTGTAATTAAGGAGATGCTTCATGACTGTCGGGTAGGCAGAG 3612
                 ********* *********** ************** ** ******** ** ******

CNSV             TACTGAGCTACATCACCCTCAGCGAGGTGGAGGGTCTTAGCACTATGGGAGGGGATAAAA 3720
CILV-N           TACTGAGCTACATCACCCTCAGCGAGGTGGAGGGTCTTAGCACTATGGGAGGGGACAAAA 3677
OFV              TACTGAGCTACATCACCCTCAGCGAGGTAGAGGGTCTCAGCACCATGGGAGGTGACAAAA 3672
                 **************************** ******** ***** ******** ** ****

                                                       IGR3
CNSV             GCCACAGTTATCGTTAAGTATGACTCAGGCGAAGTACCCTGGAGAAGAATCCCGCCTATC 3780
CILV-N           GCCACAGTTATCGTTAAGTGTGACTCAGGCGAAGTACCCTGGAGAAGAATCCCGCCTATC 3737
OFV              GCCACAGCTATCGTTAATTGTTACTCGGGCTAAGGACCCTGACGGAGAATGCTGCCTTCC 3732
                 ******* ********* * * **** *** *** *** ******   * ***** * ****  *

CNSV             CAATAAACATGTCTACTTACCTACCATAATTTAAATAAAAAAAAATCATCGAATCATTG 3840
CILV-N           CAATAAACATGTCTACTTACCTACCATAATTTAAATAAAACAACAATCATCGAATCATTG 3797
OFV              CAATAAGCATATCTACTTATCTACCGTAATTTAAATAAAACAACAATCTTCGAATCATTG 3792
                 ****** *** ******** ***** ************** ** **** ***********

                                                                  ORF4:    M
CNSV             ATTATTCATATATCGGATCTAACAATTGTCTTTTGTCATGTCAAAACAGATTAATATGTG 3900
CILV-N           ATTATTCATATATCAGATCTAACAATTGTCTTTTGTCATGTCTAAACAGATTAATATGTG 3857
OFV              ATCATTTATATATCAGATCTAACGATTGTCTTTTATCATGTCTAAGCAGATCAATATGTG 3852
                 ** *** ******* ******** ********** ****** ** ***** ********

CNSV             CACCGTGATGTCCTTCAGGTCACTAATCACTGTGATAGGACCCAGATCAGTGGATCTTGG 3960
CILV-N           CACTGTGATGTCCTTCAGGTCCCTAATCACCGTGATAGGACCCAGAGCAGTGGATCTTGG 3917
OFV              CACTGTGATGTCCTTCAGATCACTCATCACTGTGATAGGACCCAGGTCAGTGGATCTTGG 3912
                 *** ************** ** ** ***** **************  *************

CNSV             AGCCGGGTTCAAGGAGGCGGTGCTGCGTGCTATCACTTCACTCAAGATCAAGGCTAAGGA 4020
CILV-N           AGCCGGGTTCAAGGAGGCGGTGCTGCGTGCTATCACTTCACTCAGGATCAAGGCTAAGGA 3977
OFV              AGCCGGATTCAAGGAGGCGGTGCTGCGAGCAATCACTTCACTCAAGGTCAAGGCCAAGGA 3972
                 ****** ******************** ** ************* * ******* *****

CNSV             CCCAAAAGGGACAGAGATTGATGGTCCCTGGCAGGAGCTGCTGGTGGAGATTGCCACTAT 4080
CILV-N           CCCCAAAGGGACAGAGATTGAGGGTCCCTGGCAGGAGCTGCTGGTGGAGATTGCCACTAT 4037
OFV              CCCGAAAGGGGCCGAGATAGATGGCCCCTGGCAAGAGCTACTGGTGGAGATTGCCACCAT 4032
                 *** ****** * ***** ** ** ******** ***** ***************** **

CNSV             GACAAAGTCAGCATTCACCGGCCCTACCATCAAGAAAGACCTGGAGCAGGGGAATGTCTA 4140
CILV-N           GACAAAGTCAGCATTCACCGGCCCTACCATCAAGAAAGACCTGGAGCAGGGGAATGTCTA 4097
OFV              GACCAAATCAGCATTCACCGGCCCGACCATCAAGAAAGATCTAGAGCAGGGGAATGTCTA 4092
```

```
                *** ** ***************** ************** ** *****************

CNSV            CCGTTATGCAATGACAGTTGGCGGCATGTTGAGCACCTCACACAACACAAACGGGAGGCT 4200
CILV-N          TCGTTACGCAATGACAGTTGGCGGCATGTTGAGCACCTCACACAACACAAACGAGAGGCT 4157
OFV             CCGATATGCGATGACAGTGGGCGGCATGTTGAGCACATCACACAATACTAATGGAAGATT 4152
                 ** ** ** ******** ***************** ******** ** ** *  **  *

CNSV            GATGACTGTCCGGACTGGACCACTATATGATACTGACCACTACCATGTGTCGGGGGAGGT 4260
CILV-N          GATGACTGTCAGGACTGGACCACTATATGATACTGACCACTACCATGTGTCGGGGGAGGT 4217
OFV             GATGACTGTCAGGACTGGACCTCTATATGATACAGACCACTATCATGTGTCTGGGGAGGT 4212
                ********** ********** *********** ******** ******** ********

CNSV            TGATATCGACAAAATGGATGGTTTCAAGGCTTCGATAACATTATCTGTGGCCACTGTTAA 4320
CILV-N          TGATATCGACAAAATGGATGGTTTCAGAGCTTCGATAACATTATCTGTGGCCACTGTTAA 4277
OFV             TGATATTGATAAAATGGATGGTTTCAAAGCAACGATAACACTGTCTGTGGCCACTGTCAA 4272
                ****** ** ****************  **  ******** * ************** **

CNSV            GAGAGAACGTCAACAGTGCGAGGCAGGCATCGGGGAATACCATGTTTTGCCGATCAAGGG 4380
CILV-N          GAGAGAACGTCAACAGTGCGAGGCAGGCATCGGGGAATACCGTGTTTTACCGATCAAGGG 4337
OFV             AAGAGAACGTCAACAGTGCGAGGCAGGCATCGGGGAATACCATGTTTTACCGATCAAGGG 4332
                 **************************************** ****** ***********
                                                                   IGR4
CNSV            CAAAAATCCCCCACGATCCTCCAGCAACACCGCGGGACCCAGTGGTTAGAGCCATCAACA 4440
CILV-N          CAAAAATCCCCCACGATCCTCCAGCAACACCGCGGGACCCAGTGGTTAGAGCCATCAACA 4397
OFV             CAAAAATCCCCCACGATCCGCCAGCAACACCGCGGGACCCAGTGGTTAGAGCCATCAACA 4392
                ******************* ****************************************

CNSV            GCTCAGGGCCTGCTCTCCCTCCTATCAGACGTGGATAATTACTTACGGAGGAAGAAGTCT 4500
CILV-N          GCTCAGGGCCTGCTCTCCCTCCTATCAGACGTGGATGATTACTTACGGAGGAAGAAGTCT 4457
OFV             GCTCAGGGCCTGCTCTCCCTCCTATCAGGCGTGGATGATTACTTAAGGAGGAAGAAGTCT 4452
                **************************** ******* ******** **************

CNSV            CGACTGTCTTATGTTCTGAAGGACGGTGAATGAAACATATAATCATATACATGTCTTAAT 4560
CILV-N          CGACTATCCTATGTTTTGAAGGACGGTGAATGAAACATATAATCATATACATGTCTTAAT 4517
OFV             CGACTATCTTATGTTTTAAAGGACGGTGAATGAAACATATAACCATATGCATATCTTAAT 4512
                ***** ** ****** * ************************ ***** *** *******
                                                             ORF 5: G
CNSV            GATATGATCTATATAACCTGATTTAAATAAAACAATAATGATGGCACAGACAAACCAGCC 4620
CILV-N          GATATGATCTATATACCCTTATTTAAATAAAACAATAATGATGGCACCGACAAACCAGCC 4577
OFV             GATATGCCCTATATAATTTGATTTAAATAAAACAATAATGATAGTACAAACGAAACAGCC 4572
                ******  *******   * ********************** * **  ** ** *****

CNSV            CGACACTATGTCAATCTTCCGATCTTCAATCATATTAATGCTTTTCCTTATCTCAGGGTC 4680
CILV-N          CGACACTATGTCAATCTTCCGCTCCTCAATCATATTAATGCTTTCCCTTATCTCAGGTTC 4637
OFV             CGGCACGATGTCAACCTTCTGCTCTTTAACCGTATTAATGTTTCTTCTTATCTCAGGTTT 4632
                ** *** ******* **** * ** * ** * ******** **   *********** *

CNSV            TGGAGCACTATCCTTGGTTCCTAAGACAGTTTGTGAAAAAGAGGTGGGGCTGCATGTGGA 4740
CILV-N          CGGAGCGCTATCCTTGGTTCCTAAGACAGTTTGTGAGAAAGAGGTGGGGCTGCATGTGGA 4697
OFV             TGAGGCTTTGTCCCTGGTCCCAAAGACAGTTTGTGAAAAAGAGGTGGGGCTACATGTGGA 4692
                 *  **  * *** **** ** ************** ************** ********

CNSV            TGAATGGCTCCACTCATGTTTGGGGGCATGCAAGAGCAGCGACAACGATCTAGATATGAC 4800
CILV-N          TGAATGGCTCCACTCATGTTTGGGAGCATGCAAGAGCAGCGACAACGATCTAGATCTGAC 4757
OFV             TGAATGGCTCCATTCATGTTTGGGGGCCTGCAAGAGCAGCGATAACGACCTTGATATGAC 4752
                ************ *********** ** ************** ***** ** *** ****

CNSV            TCCACACATGTTAATGGAGCCCACATTGGGGTATTTCAAGGCCTTCGGGTATTTCATACA 4860
CILV-N          TCCACACATGTTAATGGAGCCCACATTGGGGTATTTCAAGGCCTTCGGATACTTCATACA 4817
OFV             ACCCCACATGTTGATGGAGCCCACATTAGAGTACTTTAAGGCCTTCGGGTACTTCATACA 4812
                 ** ******** ************** * *** ** *********** ** ********

CNSV            TGTATCAACCCTTACCAAATCTTCACACACTTTCCTGTTCGGCGGGTGTCACATCACATC 4920
CILV-N          TGTGTCAACCCTTACCAAATCTTCACACACTTTCCTGTTCGGAGGGTGTCACATCACATC 4877
OFV             TGTATCAACCCTCACCAAGTCGTCACATACCTTCCTACTCGGTGGGTGTCACATCACCTC 4872
                *** ******** ***** ** ***** ** *****  **** ************** **


CNSV            ACAAGAGACTCCTAGTGATTCTCAGGAGCTTCCCACCAATATGGCCGAGAATATACTGAG 4980
CILV-N          ACAAGAGACTCCTAGTGATTCTCAGGAGCTTCCCACCAATATGGCCGAGAATATACTGAG 4937
OFV             ACAGGAGACCCCCAGCGATTCTCAAGAGCTCCCCACTAATATGGCTGAGAATATATTGAG 4932
```

```
                 *** ***** ** ** ******** ***** ***** ******** ********* ****

CNSV             ACATGGCGGTCCTGAGGGAGAGGTTTTTATGACGAAAGAGCCCCAATGCAGCCTCTGGAG 5040
CILV-N           ACATGGCGGCCCTGAGGGAGAGGTATTTATGACAAAAGAGCCCCAATGCAGCCTCTGGAG 4997
OFV              GCATGGCGGCCCTGAGGGAGAGATATTTATGACAAAAGAGCCCCAATGCAGCCTCTGGAG 4992
                  ******** ************ * ******** **************************

CNSV             TGACAATTATGTGAAAGGCATGCTTGTGAAGTATCACAGGGTGATTTTGACTGTCTCGCA 5100
CILV-N           CGACAATTATGTGAAAGGCATGCTTGTGAAGTATCACAGGGTGATTTTGACTGTCTCGCA 5057
OFV              TGACAATTATGTGAAAGGCATGCTTGTGAAGTATCACAGGGTGATTTTGACTGTCTCACA 5052
                  ******************************************************** **

CNSV             CACCGAAAGAGGGATAACGGTTCTTTACGAGCAGGAGGGAGTGACGGGCCATGGCAAGGT 5160
CILV-N           CACCGAAAGAGGGATAACGGTTCTTTACGAGCAGGAGGGAGTGATAGGCCACGGCAAGGT 5117
OFV              CACCAAGAGCGGGATAACGGTTCTTTACGAGCAGGAGGGAGTGATAGGCCATGGCAAGGT 5112
                 **** * ** **********************************  ***** ********

CNSV             CGGTAAAACCGTGTCATCATCCGGTACTCTCGTGTGGGACATACGGGCACAATACCCAAA 5220
CILV-N           CGGTAAAGCCATGTCATCATCGGGTACTCTCGTGTGGGACATACGGGCACAATACCCAAA 5177
OFV              CGGCAAAACCACTTCATCATCAGGAACCCTTGTGTGGGACGTGAGAGCACAATACCCGAA 5172
                 *** *** **   ******** ** ** ** ********* *  * *********** **

CNSV             GTGCAGCTATAGACCTACGGGGGTTCTTAGCTGTAAACGAGAGGGATCCTACATACGTTG 5280
CILV-N           GTGCAACTATCGACCTACGGGGGTTCTTAGCTGTAAACGAGAGGGATCCTACATACGTTG 5237
OFV              GTGCAACTATCGCCCTACAGGGGTTCTTAGCTGTAAGAGAGAAGAATCATACATACGTTG 5232
                 ***** **** * ***** *****************  **** * *** ***********

CNSV             CAGAGGTATGTCGGAGGAGAGAATCTCATCCACCAAGGAGGACTGCGGGGTCACCGTATT 5340
CILV-N           CAGAGGTATGTCAGAGGAGAGAATCTCATCCACCAAGGAGGACTGCGGGGTCATTATATT 5297
OFV              CAGAGGTATGTCAGAGGAGAAAATTTCATCCATCACGGAGGACTGTGGAGTCATCATATT 5292
                 ************ ******* *** ******* ** ********* ** ****   ****

CNSV             AACGACGGACACAAGGAATATATATGGGTACCATAAACATGATGGTGCACTGGCCACCAC 5400
CILV-N           AACGACGGACACAAGGAATATATACGGGTACCATAAACATGATGGTGCATTGGCCACAAC 5357
OFV              AACGACAGACACAAGGAACATATATGGATACCATAAACATGATGGAGCATTGACTACCAC 5352
                 ****** *********** ***** ** ***************** *** ** * ** **

CNSV             TCAGACGGATGACAATCAGATAGGCATGGTGAAGAAGATCATAGAGATAGAGAATCTCAT 5460
CILV-N           TCAGACGGATGACAATCAGATAGGTATGGTGAAGAAGATCATAGAGATAGAGAATCTTAT 5417
OFV              TCAGACGGATGACAATCAGATAGGTATGGTGAAGAAGATCATCGAGATAGAGAATCTCAT 5412
                 ************************ ***************** ************** **

CNSV             GTGTCATCATCTATGTGAGTCCTCATCCGAGGAGGGGGGAGTTACACACCATGAATATTT 5520
CILV-N           GTGTCATCATCTATGTGAGTCCTCATCTGAGGAGGGGGGAGTGACACACCATGAATATTT 5477
OFV              GTGTCATCATCTATGTGAGTCCTCATCTGAGGAAGGGGGAGTGACACACCATGAATATTT 5472
                 *************************** ***** ******** *****************

CNSV             GGTTTCCACTCCAATAGGACCATGGCTATCCGTATCCACAGAGTCCCATCGCACCATGTT 5580
CILV-N           GGTCTCCACTCCTATAGGACCATGGCTATCTGTATCCACAGAGTCCCATCGCACCATGTT 5537
OFV              GGTCTCCACACCGATAGGGCCGTGGCTAGCCGTCTCTACGGAGTCCCATCGCACCATGTT 5532
                 *** ***** ** ***** ** ****** * ** ** ** ********************

CNSV             CATGTGTACAGATGAAAGCACAGTGTCATTGGTGATTCCTGTGGTACTATGTGGCAATGG 5640
CILV-N           CATGTGTACAGATGAAAGCATAGTGTCACTGGTGGTTCCTGTGGTACTATGTGGCAATGG 5597
OFV              TATGTGTACGGATGAGAGTGCAGTGTCATTGGTGGTTCCAGTGGTATTGTGTGGCAATGG 5592
                  ******** ***** **   ******* ***** **** ****** * ***********

CNSV             ACCCTTGGTGAAAGTAAGGGTCAATGAGAAGGAACAATGGTGGAATATCTCCTCTCCGTA 5700
CILV-N           ACCCTTGGTGAAGGTAAGGGTCAATGAGAAGGAACAATGGTGGAATATCTCCTCTCCGTA 5657
OFV              GCCCTTGGTGAAAGTAAGGGTTAATGAGAGGGAGCAATGGTGGAACATATCCTCTCCATA 5652
                  *********** ******** ******* *** *********** ** ******** **

CNSV             TGTTGGCATGGGGACTCATTGTGTCCCAGGATTGTCTACAGGATTATCTACTGCTGATGG 5760
CILV-N           TGTTGGCATGGGGACTCATTGTGTCCCAGGCTTGTCTACAGGCTTATCTACTGCTGACGG 5717
OFV              TGTTGGTATGGGGACTCATTGTGTCCCGGGCCTGTCTACTGGATTATCCACTGTTGATGG 5712
                 ****** ******************** **  ******* ** ***** **** *** **

CNSV             TGTGATAAACACATGGTTAGGACAAGTCACCATAGTGAATGGATCCTTCACTTTCAAGCG 5820
CILV-N           TGTGATAAACACATGGTTAGGACAAGTCACCATAGTGAATGGATCCTTCACTTTCAAGCG 5777
OFV              TGTGATAAACACATGGTTGGGACAAGTCACCATAGTGAACGGATCTTTCACTTTCAAGCG 5772
                 ****************** ******************** ***** **************
```

```
CNSV         TGATTTTATCAATAAGAATTTCCATGGGGCTTTCAGGCCAAGCCAGTTTAGCTGGTCCCA 5880
CILV-N       TGATTTCATCAATAAGAATTTCCACGGGGCTTTCAGGCCAAGCCAGTTTAGCTGGACCCA 5837
OFV          TGATTTTATCAATAAGAACTTTCACGGGGCTTTCAGGCCAAGCCAGTTTAGCTGGTCTCA 5832
             ****** *********** ** ** ****************************** * **

CNSV         CAAAACAGGCAATGATCTTGAACACATCATAGATGCTCTGAATACACAGGAGAAAGTCCT 5940
CILV-N       CAAAACAGGCAATGATCTTGAACACATCATAGATGCTCTGAATACACAGGAGAAAGTCCT 5897
OFV          CAAGACAGGCAATGATCTTGAACACATCATAGATGCTCTTAACACACAGGAGAAAGTCCT 5892
             *** *********************************** ** *****************

CNSV         AAGCCACTCTCATGTGATAGAATCACACAGTGTGGGAGCTGGAGAGAACGCGTTGCACTC 6000
CILV-N       AAGCCACTCTCATGTAATAGAATCACACAGTGTGGGAGCCGGAGAGAACGCGTTGCACTC 5957
OFV          AAGCCACTCTCATGTGATAGAATCACACAGTGTGGGGGCCGGGGAGAATGCTGTGCACTC 5952
             *************** ******************** ** ** ***** **  *******

CNSV         GTTCATCGGTATGTTCACGACTGTATTCGAATGGATACAGAACTTAATCCCAAATGTCAA 6060
CILV-N       GTTCATTGGTATGTTCACGACTGTATTCGAATGGATACAGAGCTTAATCCCAAGTGTCAA 6017
OFV          GTTCATCGGTATGTTCACGACTGTGTTCGAGTGGATACAAAGCTTGATCCCAAATGTCAA 6012
             ****** ***************** ***** ******** * *** ******* ******

CNSV         AGGGTGGATCATCAAAATCTTCCTGTGGGCATTGTTGGCAGCCCTGGCAGTATTGTTACT 6120
CILV-N       AGGGTGGATCATCAAAGTCTTCCTGTGGGCATTGTTAGCAGCTCTGGCAGTATTGTTACT 6077
OFV          AGGGTGGATAATCAAGATCTTCCTGTGGGCACTGTTGGCAGCCCTGGTAATCCTGTTGCT 6072
             ********* *****  ************** **** ***** **** * *  **** **

CNSV         ATGGATATTGTGGAAGGTATTATGGTTGTTCATCAAATCTGTGTTTTTAAGGCAGTCTAT 6180
CILV-N       ATGGATATTATGGAAGATATTATGGTTGTTCATCAAATCTGTGTTTGTAAGGCGGTCTAT 6137
OFV          CTGGATATTGTGGAAGGTATTATGGTTGTTCATCAAATCTGTATTCCTAAGGCGGACCAT 6132
              ******** ****** ************************* **  ****** * * **

CNSV         TCGGGCAATCCCAACATCGGAAAATTCAGACACATCACTGAATCGGGCCATCCACAATTG 6240
CILV-N       TCGGGCAATCCCAACATCGGAAAATTCAGACACATCACTGAATCGGGCCATCCACAATTG 6197
OFV          TCAGGCAGTCCCAACATCGGAGAACTCAGACACATCGCTGAATCGGGCCATCCACAACTG 6192
             ** **** ************* ** *********** ******************** **

                               Trailer
                               ------------------------------>
CNSV         GGCCAAGATGGATTAACAAGGTCAGTTCGGGGCTCTTCACATATATACATATAGCTAATA 6300
CILV-N       GGCCAAGATGGATTAACCAGGTCAGTTCGGGGCTCTTCATATATATATATATAGCTAATA 6257
OFV          GGCCAAGATGGATTAACAAGGTCAGTTAGGGACTTTTTATATATATACATATAGCTGATA 6252
             ***************** ********* *** ** ** * ******* ******** ***

             ------------------------------------------------------------>
CNSV         AATTTAAATAAAACTACCCCTCTAGGGTCTTTGTCTAGTTAGGGATGTAGGGGAAAGGAC 6360
CILV-N       AATTTAAATAAA------------------------------------------------ 6269
OFV          AATTTAAATAAAACTATCTCTTGGGGGCCTTTTCCTAGTTAGGGATGTAGGGTAAAGGAC 6312
             ************

             ------------------------------------------------------------>
CNSV         CAATAGAGTCGCATAAGAGATCGTAGCGTTCGTCAACATCATCAACGTTGGTCGATGCGG 6420
CILV-N       ------------------------------------------------------------
OFV          CAATAGAGTCGCATAAGAGATCGTGGCGTTCGTCAACATCATCAACGTTGGTCGATGCGG 6372

             ------------------------------------------------------------>
CNSV         GATATGTTGACTATTTACAACAGTTACTCATGGCTCTGTGTCCCATGTACTCTGCGTTGA 6480
CILV-N       ------------------------------------------------------------
OFV          AATATGTTGACTATTTACAACAGTTACTCATGGCTCTGTGT------------------- 6413

             --------------->
CNSV         TACCACTGCTTAGAT 6495
CILV-N       ---------------
OFV          ---------------
```

Comparison of RNA2.

```
                 Leader
CNSV       GAGGAGTCATGGGGACACAGGACAACCAACTGTCTCATTGCGAAGTCAAATTCCGTGATG 60
CILV-N     --------------ACACAGGACAACCAACTGTCTCATTGCGAAGTCAAATTCCGTGATG 46
OFVRNA2    --------------ACACAGGACAACCAACTGTCTCATTGCGAAGTCAAATTCCGTGATG 46
                         **********************************************

CNSV       GGTCCGTCTTTCCTAGAATAAGGGAAGGATACTATAGGATTGATCCTAGTTCTTCTCTAC 120
CILV-N     GGTCCGTCTTTCCTAGAATAAGGGAAGGATACTATAGGATTGATCCTAGTTCTTCTCTAC 106
OFVRNA2    GGTCCGTCTTTCCTAGAATAAGGGAAGGATACTATAGGATTGATCCTAGTTCTTCTCTAC 106
           ************************************************************

CNSV       GCTGGGTGCTACACGGATCATGCAAAACAACCAAATATCATATATTATTGCACATACACT 180
CILV-N     GCTGGGTGCTACACGGATCATGCAAAACAACCAAATATCATATATTATTGCGCATACACT 166
OFVRNA2    GCTGGGTGCTTCACGGATCATAAAAAACAACCAACCATCATATATTATTACGCATACACT 166
           ********** **********  ***********  ************* * ********
                                   ORF 6: L
CNSV       TTTGAACTAATCAGATATGAATTCACTATTTAAGCAGTGGTATCAACGCAGAGTACT-AA 239
CILV-N     TTTGAACTAATCAGATATGAATTCACTGTTTCAGGAATGTTA----CACAGAGTCCTTAA 222
OFVRNA2    TTTGAACTAATCAGATATGAATTCACTATTTCAGGAATGTTA----CACCGAGTCCCTGA 222
           *************************** *** ** * ** **    * * **** *   *

CNSV       TGGGCGATAACACGGACACCGTCATCACCGGTAAGGGGAGATCCGCCCATAACGAGGGAG 299
CILV-N     TGGACGATAACACGGACACCATCATCACCGGTAAGGGGAGATCTGCCCATAATGAGGGAG 282
OFVRNA2    TGGACGATAATACGGACACCATCATCACCGGTAAGGGGAGGTCTGCCCACAATGAGGGAG 282
           *** ****** ********* ******************* ** ***** ** *******

CNSV       AATACCATCTAAAGTCTGCCTTGAGAAGCCACAGTGATTATGTGAAGAACAAGCATCAAG 359
CILV-N     AATACCATCTAAAGTCTGCCCTGAGGAGCCACAGTGATTATGTGAAGAACAAGCATCAAG 342
OFVRNA2    AATACCACCTAAAGTCTGCCCTGAGAAGCCACAGTGATTATGTGAAAAACAAGCATCAAG 342
           ******* ************ **** ******************** *************

CNSV       CCAAGGATCTGCTGAAGCTCAGTGAGCATACATCCGTATATGCATCAAAGAATCCATGCA 419
CILV-N     CCAAGGATCTGCTAAAGCTCAGTGAGCATACATCCGTACATGCATCGAAGAATCCATGCA 402
OFVRNA2    CCAAGGATCTGCTAAAGCTCAGTGAACATACATCTGTATATGCACCGAAAAACCCATGCA 402
           ************* *********** ******** *** ***** * ** ** *******

CNSV       AATTGCTACCCACTCTATGGAAGGGAGTGATCGAGGGGGAGGAGTGGGTTGAAGATGAGG 479
CILV-N     AACTGCTACCCACTCTATGGAAGGGAGTGGTCGAAGGGGAGGAGTGGGTCGAGGATGAGG 462
OFVRNA2    AGTTACTGCCCACTCTATGGAGAGGGGTGATTGAAGGGGAGGAGTGGGTTGAAGATGAGG 462
           *  * ** *************  ** *** * ** ************** ** *******

CNSV       TTGTAATATATCTAAAAGAGCTCTATCATGATGACGAGACTGATGATTGTGCTCTTGAGC 539
CILV-N     TTGCAATATATCTAAAGGAGCTCTATCATGATGACGAGACTGATGATTGTGCTCTTGAGC 522
OFVRNA2    TTATAATATACTTGAGAGAGCTCTATCATGACGACGAAACAGACGACTGTGCTCTTGAGC 522
           **  ******  * *  ************** ***** ** ** ** *************

CNSV       TCCTCGATGCAGAGCTGGAGCATGTGTGCCACGATGATGACAAAACAGGGATAATGCAGG 599
CILV-N     TCCTCGATGCAGAGCTGGAGCATGTGTGCCACGACGATGACAAAACAGGGATAATGCAGG 582
OFVRNA2    TCCTTGATGCAGAGCTGGAACATGTGTGTCATGACGATGACAAAACAGGGATAATGCAGG 582
           **** ************** ******** ** ** *************************

CNSV       GGGTCGTACAGTGTCAGGAGGATGCATGGAACAAACACACCATCTACGGTAGAGCTAATA 659
CILV-N     GGGTCGTACAGTGTCAGGAGGATGCATGGAACAAACACACCATCTACGGTAGAGCTAACA 642
OFVRNA2    GAGTCGTACAGTGTCAGGAGGATGCATGGAACAAACATACCATCTACGGTAGGGCCAATA 642
           * *********************************** ************** ** ** *

CNSV       TCCTCCGGTGGGCCCTGACAAGGATAGTGATAGATCATAATAAAGCAGTGGTATCAACGC 719
CILV-N     TCCTTCGGTGGGCCCTGACAAGGATAGTGATAGATCATAATAAAGAGGAGCCAACAGAGC 702
OFVRNA2    TCCTTCGATGGGCCTTGACAAGGATAGTGATCGATCACAATAAAGAGGAGCCTACAGAAC 702
           **** ** ****** **************** ***** *******  * *    **   *

CNSV       AGAGTACCATGGGGAGAAAGTTGGATGATAAGGTGGTTGCTTATTCATTGAGCAGCTCAT 779
CILV-N     TTAGTCCCATGGGGAGGAAGTTGGATGGCGGGGTGTTTGCTTATTCATTGAGCAGCTCAT 762
OFVRNA2    TCAGCCCCATGGGGAGGAAATTGGCCGATAAGGTGTTTGCTTATTCACTGAGCAGCTCAT 762
             **  ********** ** ****  *    **** *********** ************
```

Cruz-Jaramillo *et al.*, 2014.

```
CNSV        TATCTATGATGATAAGCAGAAGTTGGTGTCATCTGATGATCAATGACAGCCCCGGAACCA 839
CILV-N      TATCTATGATGATAAGCAGAAGTTGGTGTCATCTGATGATCAATGACAGCCCCGGAACCA 822
OFVRNA2     TATCTATGATGATAAGCAGAAGTTGGTGTCATCTGATGATTAATGACAGCCCAGGCACCA 822
            **************************************** *********** ** ****

CNSV        TCAACATTCAGCACCTGATCAACACCCTGGATAAACTGACAGAACGAGTGAACATAATGA 899
CILV-N      TCAACATTCAGCATCTGATCAACACCCTGGATAAACTGACAGAACGAGTGAACATAATGA 882
OFVRNA2     TCAACATCCAGCACTTGATCAACACCCTGGATAAACTGACGGAACGAGTGAACATAATGA 882
            ******* *****  ************************* *******************

CNSV        CTTATCTAACCCTCTGCAACAAGACTGGGCTCTCAAGCATTTATCCGTCAGTTGACATCA 959
CILV-N      CTTATCTAACCCTCTGCAACAAGACTGGGCTCTCAAGCATTTATCCGTCAGTTGACATCA 942
OFVRNA2     CCTACCTAACTCTCTGCAATAAGACTGGACTTTCAAGCATTTATCCATCAGTAGACATCA 942
            * ** ***** ******** ******** ** ************** ***** *******

CNSV        CTCTGAGAATCATTCAGCTAGGTGACCAATTGATACTGAATGAGGGCAATAATGCCTTTC 1019
CILV-N      CTCTGAGAATCATTCAGCTAGGTGACCAATTGATACTGAATGAGGGCAATAATGCCTTTC 1002
OFVRNA2     CTTTGAAAATAATTCAGCTGGGTGACAATTTAATATTAAATGAAGGCAATGATGCTTTCC 1002
            ** *** *** ******** ****** * ** *** * ***** ****** **** ** *

CNSV        GCGGGCTGGCCAGTTATGAAGCTATCTGTGTCGGTGAGATAATCAATAGGGGGGACTCAT 1079
CILV-N      GCGGGCTGGCCAGTTATGAAGCTATCTGTGTAGGTGAGATAATCAACAGGGGGGACTCAT 1062
OFVRNA2     GCGGGTTGGCCAGTTACGAAGCCATCTGTGTCGGTGCGATAATCAACAGGGGCGATTCGT 1062
            ***** ********** ***** ******** **** ********* ***** ** ** *

CNSV        CAGTGTGGGATGTCGGGAGATTTCTCAACACCATGCTACAGGAGGTTGGTGCATTGGGAG 1139
CILV-N      CAGTGTGGGATGTCGGGAGATTTCTCAACACCATGCTACAGGAGGTTGGTGCATTGGGGG 1122
OFVRNA2     CTGTGTGGGATGTTGGTAGATTCCTCAACACTATGGTACAAGAGGTTGGATCATTGGGAG 1122
            * *********** ** ***** ******** *** **** ********  ******* *

CNSV        AGAACTACAAGACATGGTCATCAGATGTTCTGGTAATACTTCAAGGGCTAAGGATACAAC 1199
CILV-N      AGAACTACAAGACATGGTCATCAGATGTTCTAGTAATACTTCAAGGTCTAAGGATACAAC 1182
OFVRNA2     AGAACTACAAGACATGGTCATCTGATGTCCTGGTGATACTGCAAGGTTTGAAGATCCAGC 1182
            ********************** ***** ** ** ***** *****  * * *** ** *

CNSV        AAGTGTCATGCCTGCATGGCATATATCGCATCTGGGGACACCCGGTTGTTGATCTTGAGA 1259
CILV-N      AAGTGTCATGCCTGCATGGCATGTATCGCATCTGGGGACATCCGGTTGTTGATCTTGAGA 1242
OFVRNA2     AAGTGTCATGCCTGCATGGCATGTATCGTATCTGGGGACATCCAGTTGTTGATCTTGAGA 1242
            ********************** ***** *********** ** ****************

CNSV        AAGGCCTCATGAAGCTCCGCTCGGTGGCTTTGATGGAGAAAGAGATTCCGCCTGAATGTG 1319
CILV-N      AAGGCCTCATGAAGCTCCGCTCGGTGGCTCTGATGGAGAAAGAGATTCCGCCTGAATGTG 1302
OFVRNA2     AGGGCCTCATGAAGCTTCGTTCAGTAGCTCTAATGGAGAAGGAGATCCCACCAGAATGTG 1302
            * ************** ** ** ** *** * ******** ***** ** ** *******

CNSV        CCAGAAATACCTCCAATATGTTCAAGGAGATATTCTTCACACAGTATTTCAGGCGCCACA 1379
CILV-N      CCAGGAATACCTCCAACATGTTCAAGGAGATATTCTTCACACAGTATTTCAGGCGACACA 1362
OFVRNA2     CCAGGAATACCTCCAATATGTTCAAGGAGATATTCTTCACGCAATACTTCAGACGCCACA 1362
            **** *********** *********************** ** ** ***** ** ****

CNSV        AGTTTTACCCTCCACATACCTGGACCGGTCCTGAAGGAGGCAATTACATCAAGCAGGTCC 1439
CILV-N      AGTTTTACCCTCCACATACCTGGACTGGTCCTGAAGGAAGCAATTACATCAAGCAGGTCC 1422
OFVRNA2     AGTTTTACCCCCCACACACCTGGACCGGTCCGGAAGGAAGCAATTACATCAAGCAGGTCC 1422
            ********** ***** ******** ***** ****** *********************

CNSV        TATCACTTGAGAAAGAGTTGGATATACACAACATAAGATATCACTTGGAGGACTGGGGGA 1499
CILV-N      TATCACTTGAGAAAGAGTTAGATATACACAACATAAGATATCACTTGGAGGACTGGGAGA 1482
OFVRNA2     TATCACTTGAGAAAGAGTTGGATATACACAACATAAGATACCACTTGGAGGATTGGGAGA 1482
            ******************* ******************** *********** **** **

CNSV        ATATCAGATGTGAGAAGACATTCGAGATCCCGGCCACATACAGCCTTGCATCATGTATCA 1559
CILV-N      ATATCAGATGTGAGAAGACATTCGAGATCCCGGCCACATACAGCCTTGCATCATGTATCA 1542
OFVRNA2     ACATCAGATGTGAGAAGACCTTCGAGATTCCGGCCACATACAGTCTTGCATCATGTATCA 1542
            * ***************** ******** ************** ****************

CNSV        AAGACAGAGCAATATCCCCAAAACGATCAGAGTTGGTATCCATGATCATCAGAGGAGGAT 1619
CILV-N      AAGATAGAGCAATATCCCCAAAACGATCAGAGTTGGTATCCATGATCATTAGAGGAGGAT 1602
OFVRNA2     AAGACAGAGCTATATCCCCGAAACGGTCAGAGTTGGTATCCATGATAATCAGAGGGGGAT 1602
            **** ***** ******** ***** ******************** ** ***** ****
```

```
CNSV                CTGTGATGAGTCAAAGCTCCAGAAGGGGGGTGTTAAAATGGCTAGACAGCACCATGATCC 1679
CILV-N              CTGTAATGAGTCAGAGCTCCAGAAGGGGGGTGTTAAAATGGCTGGACAGTACCATGATCC 1662
OFVRNA2             CTGTGATGAGTCAAAGCTCAAGGAGGGGGGTTCTGAAATGGCTGGATAGTACCATGATTC 1662
                    **** ******** ***** ** ********  * ******** ** ** ******** *

CNSV                CTGTGAGAGAATTCCTGGATGGTATAAATGAGTCGGGTATGTCAAAGGACAACTCAATTA 1739
CILV-N              CCGTGAGAGAATTCCTGGATGGTATAAATGAGTCGGGATTGTCAAAGGACAACTCAATTA 1722
OFVRNA2             CTGTGAGAGAATTCTTGGATGGCATAGATAAATCAGGATTGTCAAAAGACAACTCAATTA 1722
                    * ************ ******* *** ** * ** **  ******* *************

CNSV                TTGGCCTATATCCCAAAGAACGAGAGTTGAAGATGGAGGCCAGGTTCTTTGCTCTGATGT 1799
CILV-N              TTGGCCTATATCCTAAAGAACGAGAGTTGAAGATGGAGGCCAGGTTCTTTGCTCTGATGT 1782
OFVRNA2             TCGGCCTGTACCCAAAGGAAAGAGAGTTGAAGATGGAGGCCAGGTTCTTTGCTCTAATGT 1782
                    * ***** ** ** ** *** ********************************** ****

CNSV                CTTTCCAAATGAGGTTATACTTCACAACGACAGAGTACATGGCGAATGACAACCTCCTGG 1859
CILV-N              CCTTTCAAATGAGGTTGTACTTCACAATTACAGAGCATATGGCGAATGACAACCTCCTGG 1842
OFVRNA2             CCTTTCAAATGAGGTTATACTTTACAATCACAGAGCATATGGCAAATGACAACCTCTTGG 1842
                    * ** *********** ***** ****  ****** * ***** ************ ***

CNSV                ACTACTTTCCCATGGTCACAATGTCCGACAGCATGCTGGAGCTGAACAAAAAGCTGGACA 1919
CILV-N              ACTACTTTCCCATGGTCACAATGTCCGACAGCATGCTGGAGCTGAACAAAAAGCTGGACA 1902
OFVRNA2             ACTACTTCCCCATGGTCACAATGTCTGACAGCATGCTGGAGCTAAACAAGAAGCTGGACA 1902
                    ******* ***************** ***************** ***** **********

CNSV                CTCTAAGTAGAAAGCAGGTCACAAAGAAGGAGGGTGTGGTACATTATGTGGTCAATATAG 1979
CILV-N              CTCTAAGTAGAAAGCAAATCACAAAGAAGGAGGGTGTGGTACATTATGTGGTCAATATAG 1962
OFVRNA2             CTCTGAGCCGAAAGCAGGTTACAAAAAAGGAGGGTGTGGTGCATTATGTGGTCAACATCG 1962
                    **** **  *******  * ***** ************** ************** ** *

CNSV                ATTTCAGAAAATGGAACCAACAGATGAGGGAAGAGATGACTGTCCCTATGTTTCTTGATG 2039
CILV-N              ATTTCAGAAAATGGAACCAACAGATGAGGGAAGAGATGACTGTCCCTATGTTTCTTGATG 2022
OFVRNA2             ATTTCAGAAAATGGAACCAACAGATGAGAGAGGAGATGACTGTCCCCATGTTCCTTGATG 2022
                    **************************** ** ************** ***** *******

CNSV                CTGACAGACTGTTCGGTTACACCAATCTGGTGGGGCGGACGCATGAGATCTTCAAGTCCT 2099
CILV-N              CTGATAGACTGTTCGGTTACACCAATCTGGTGGGGCGGACGCATGAGATCTTTAAGTCCT 2082
OFVRNA2             CTGATAGACTGTTTGGCTACACCAATCTGGTGGGGAGGACACATGAGATTTTCAAGTCCT 2082
                    **** ******** ** ****************** **** ******** ** *******

CNSV                CATATATATATCTATCCTCAGGGGAATACGTACCGCAAGTCTCACTCAGAGGAAGGTTGG 2159
CILV-N              CATATATATACTTATCCTCAGGGGAATACGTACCACAAGTCTCACTCAGAGGAAGGTTGG 2142
OFVRNA2             CATATATATACTTATCCTCAGGGGAATATGTACCACAAGTCTCACTCAGAGGAAGGTTGG 2142
                    **********  **************** ***** *************************

CNSV                TGCATGAGGAGCCCTACAGTTGGACCAACGACCCCTCGGGGAAAGAAGGTCTGAGGCAAA 2219
CILV-N              TGCATGAGGAGCCCTACAGTTGGACCAATGACCCCTCTGGGAAGGAAGGTCTGAGGCAAA 2202
OFVRNA2             TGCAAGAGGAGCCCTACAGTTGGACCAATGACCCTTCTGGGAAAGAGGGCCTGCGGCAGA 2202
                    **** *********************** ***** ** ***** ** ** *** **** *

CNSV                AATACTGGACCATCATGACTGCATGTGATCTGATGTATGTAGCCAGACAACACGGACTGA 2279
CILV-N              AATATTGGACCATCATGACTGCATGTGATTTGATGTATGTGGCTAGACAACATGGGCTGA 2262
OFVRNA2             AATATTGGACCATCATGACTGCGTGTGACTTAATGTATGTAGCTAGACAACATGGGTTGA 2262
                    **** ***************** *****  * ******** ** ******** **  ***

CNSV                AGATTGATCTGGTAGGCGGGGGTGACAATCAGGTCCTGATTGTGGAAGTGACCACAGACA 2339
CILV-N              AGATTGATCTGGTAGGCGGGGGTGACAATCAGGTCCTGATTGTGGAAGTGACCACAGACA 2322
OFVRNA2             AGATTGATCTGGTGGGCGGGGGAGACAATCAGGTGCTGATTGTGGAGGTGACCACCGACA 2322
                    ************* ******** *********** *********** ******** ****

CNSV                AAGTCGGGGATGGAGGTGAATTGACCACGGAAGGGAAGTCTGAGTGCAAATTCAAGATGG 2399
CILV-N              AAGTCGGGGATGGAGGTGAATTGACCACGGAGGGGAAGTCTGAGTGCAAATACAAGATGG 2382
OFVRNA2             AGGTTGGAGATGGAGGTGAATTAACAACGGAGGGGAAATCCGAGTGCAAATTCAAGATGG 2382
                    * ** ** ************** ** ***** ***** ** ********** ********

CNSV                GGACATTCATGAGCCATCTGTCCAATTACATGGAGAAGAAAGGCCTGCCGTTAAAAACAG 2459
CILV-N              GGACATTCATGAGCCATCTGTCCAATTACATGGAGAAGAAAGGCCTGCCGCTGAAAACAG 2442
OFVRNA2             AAACATTCATGCGCCATCTGTCCAATTACATGGAGAAGAAAGGCCTGCCGCTAAAGACAG 2442
                      ********* ************************************** * ** ****
```

```
CNSV       AGGAAACCTGGATTTCACCCAACCTACTCATGTTCTTCAAGATGATGTATTATGATCATA 2519
CILV-N     AGGAGACCTGGATTTCACCCAACCTACTCATGTTCTTCAAGATGATGTATTATGATCATA 2502
OFVRNA2    AGGAAACCTGGATCTCCCCCAACCTGCTCATGTTCTTCAAGATGATGTATTATGACCATA 2502
           **** ******** ** ******** ***************************** ****

CNSV       CTACTCTGGTGTCACCTCTCAAGCAAGCCTCGAGAGTGTTTCCGCTGTCAAATGATCAAG 2579
CILV-N     CTACTCTGGTGTCACCTCTCAAGCAAGCCTCGAGAGTGTTTCCGCTGTCAAATGATCAAG 2562
OFVRNA2    CCACTCTGGTGTCACCTCTCAAACAGGCCTCGAGGGTGTTTCCGTTATCTAATGATCAAG 2562
           * ******************** ** ******** ********* * ** **********

CNSV       TCATGACTATTGGCAACATGGCAGCCACTGTCTCTTCAGGGGTTACAGTACTATCCTCCA 2639
CILV-N     TCATGACTATTGGCAACATGGCAGCCACTGTATCTTCGGGGGTTACAGTACTATCCTCCA 2622
OFVRNA2    TCATGACTATTGGCAACATGGCAGCCACTGTGTCCTCAGGAGTCACAGTACTATCCTCCA 2622
           ******************************* ** ** ** ** ****************

CNSV       AGGACATGCAGATTGGCCCAGCAGCCGCATTCGGAAGACTCATATGTGCTGATTTAGCAT 2699
CILV-N     AAGACATGCAGATTGGCCCAGCAGCTGCTTTTGGAAGACTCATATGTGCTGATTTAGCAT 2682
OFVRNA2    AGGACATGCAGATTGGCCCAGCAGCTGCATTCGGAAGGCTAATATGTGCCGACTTAGCAT 2682
           * *********************** ** ** ***** ** ******** ** *******

CNSV       CGTTGGTCACCAAGGATCATCCCTTGTCTGATGGAGGAGAGATGTGGTCAGACAGCATAA 2759
CILV-N     CGCTGGTCACCAAGGATCATCCCTTGTCTGATGGAGGAGAGATGTGGTCAGACAGCATAA 2742
OFVRNA2    CATTGGTCACCAAGGATCATCCCCTGTCTGATGGAGGAGAGATGTGGTCGGACAGCATAA 2742
           *  ******************** ************************* **********

CNSV       ATATTTGCAGAGGTGGCTCTAGGAGGAAGGTCAACGTTAGGCCGGACCTCAAAACTCCGA 2819
CILV-N     ATATTTGCAGAGGTGGCTCTAGGAGGAAGGTCAACGTTAGGCCGGACCTCAAAACTCCGA 2802
OFVRNA2    GCATTTGCAGGGGAGGCTCAAGGAGGAAGGTCAACGTTAAGCCGAACCTCAAAACCCCAA 2802
             ******** ** ***** ******************* **** ********** ** *

CNSV       TGAGAATTTTCCTGTCATTAACACTCCTGCACAAGGTCATGGGAGGCCCAGCCATAGTCT 2879
CILV-N     TGAGGATTTTCCTGTCATTAACACTCCTGCACAAGGTCATGGGAGGTCCAGCCATAGTAT 2862
OFVRNA2    TGAGAATCTTCCTGTCATTAACACTCCTACACAAAGTCATGGGAGGTCCAGCCATAGTAT 2862
           **** ** ******************** ***** *********** *********** *

CNSV       CACCCCTGGGTATGATGATGAGGGGATTTCCTGATCCGCTTTGTGAACACTTGACTTGGA 2939
CILV-N     CACCCCTGGGTATGATGATGAGGGGATTTCCTGATCCGCTTTGTGAACACTTGACTTGGA 2922
OFVRNA2    CACCCCTGGGTATGATGATGAGGGGATTTCCTGATCCACTCTGTGAGCATTTGACCTGGG 2922
           ************************************* ** ***** ** ***** ***

CNSV       TCAGTATGATAAAGACAAAATTCTCACAAGCCACTAACTTTGCTTGTATGTCCATCAATA 2999
CILV-N     TCAGTATGATAAAGACAAAATTCCCCAAGCCACTAACTTTGCTTGTATGTCCATCAACA 2982
OFVRNA2    TCAGTATGATTAAGTCAAAATTCTCACAGGCCGCTAACTTCGCTTGTATGTCTATCAACA 2982
           ********** *** ******** * ** *** ******* *********** ***** *

CNSV       CCAAGGTCCCTTGGGCTCACCTACTAGAAGACCCGGTCAGTGTCAACCATGATGCTCCCA 3059
CILV-N     CCAAGGTTCCTTGGGCTCACCTACTTGAAGACCCGGTCAGTGTCAACCATGATGCTCCCA 3042
OFVRNA2    CCAAGGTCCCTTGGGCCCATCTGCTTGAAGACCCGGTCAGTGTCAACCATGACGCTCCCA 3042
           ******* ******** ** ** ** ************************** *******

CNSV       TGCATGGGCTGGCTGTGCTTAGAAGAGAGGCAGAGCAAGCCCTTGCTAAAGCCTCTGGTT 3119
CILV-N     TGCATGGGCTGGCTGTGCTTAGAAGAGAGGCAGAGCAAGCCCTCGCTAAAGCCTCTGGTT 3102
OFVRNA2    TGCATGGATTGGCTGTACTTAGAAGAGAAGCAGAGCAAGCTCTCGCTAAAGCCTCTGGCT 3102
           *******  ******* *********** *********** ** ************** *

CNSV       ATAAGAATCGAGACTTTATGGATCTGGCCCGCTGTTGTAACAAGAGTCAGATGGAGGGAC 3179
CILV-N     ATAAGAATCGAGACTTTATGGATCTGGCCCGCTGTTGTAACAAGAGTCAGATGGAGGGAC 3162
OFVRNA2    ATAAGAATCGAGATTTTATGGACCTGGCTCGCTGTTGCAACAAGAGTCAAATGGAGGGAT 3162
           ************* ******** ***** ******** *********** *********

CNSV       TGGCTGAGGCACTATGCTCTGGCAAGAATGTGGATATAAGGGTGTTGCATGATGTCATGG 3239
CILV-N     TGGCTGAGGCACTATGCTCTGGCAAGAATGTGGATATAAGGGTGTTGCATGATGTCATGG 3222
OFVRNA2    TGGCTGAGGCACTATGCTCCGGCAAGAATGTAGATATAAGGGTGTTACATGATGTTATGG 3222
           ******************* *********** ************** ******** ****

CNSV       GGGCAAGCTTAGGAGGATATTTCAACTCCATAGCCTCCAAGGTTAACAAGACTGCCACTG 3299
CILV-N     GTGCAAGCTTAGGAGGATATTTCAACTCCATAGCCTCCAAGGTTAACAAGACTGCCACTG 3282
OFVRNA2    GTGCAAGCTTAGGAGGGTATTTCAACTCCATAGCCTCTAAGGTAAACAAGACTGCCACCG 3282
           * ************** ******************** ***** ************** *
```

```
CNSV        TACTGAGAATGAACAAGAGCTCCTCAGTCATCGCGACCATTGCGGGGCAGGAACGACACT 3359
CILV-N      TCCTGAGAATGAACAAAAGCTCCTCAGTCATCGCGACCATTGCGGGACAGGAACGACACT 3342
OFVRNA2     TCCTAAGGATGAACAAGAGCTCCTCCGTCATTGCCACCATTGCAGGACAGGAAAGACATT 3342
            * ** ** ******** ******** ***** ** ******** ** ****** **** *

CNSV        GCATGAGTTATTTTGCCGCATCATCATCAGTGCCTCATGACCTTACTCCCCTGAAATGTC 3419
CILV-N      GCATGAGTTATTTTGCCGCATCATCATCAGTGCCTCACGACCTTACTCCCCTGAAATGTC 3402
OFVRNA2     GCATGAGTTATTTTGCTGCCTCATCATCAGTGCCTCACGATCTCACTCCCCTGAGATGTC 3402
            **************** ** ***************** ** ** ********** *****

CNSV        CTACAGCCACAGCTAGGAGGTACAGAAGATTGAGCTGGGGCAAGGAAATCCTGTCCATAA 3479
CILV-N      CGACAGCCACAGCTAGGAGGTACAGAAGATTGAGCTGGGGCAAGGAAATCCTGTCCATAA 3462
OFVRNA2     CAACAGCCACAGCTAGACGATACAGAAGACTGAGCTGGGGCAAGGAAATCCTGTCCATCA 3462
            * **************  * ********* **************************** *

CNSV        CTACCCCGCACCCTGCAGCCTTCCTGAAACCAGTGATTGGAGACCATCAATGTGACCACA 3539
CILV-N      CTACCCCGCATCCTGCAGCCTTCCTGACACCGGTGATTGGAGACCATCAATGTGACCACA 3522
OFVRNA2     CCACCCCGCATCCTGCAGCCTTCTTGAAACCAGTGATAGGAGACCATCAGTGTGACCACA 3522
            * ******** ************ *** *** ***** *********** **********

CNSV        ACTATGTGCAGACCAAGACATCAGGAGGGAACCTGATCAACCCACACTCTAGAGGACCAT 3599
CILV-N      ACTATGTGCAGACCAAAACATCGGGAGGGAACCTGATCAACCCACACTCTAGAGGGCCAT 3582
OFVRNA2     ACTATGTGCAGACCAAGACATCAGGAGGGAACCTGATCAATCCCCACTCTAGAGGGCCGT 3582
            **************** ***** ***************** ** *********** ** *

CNSV        ACCCGGTTTATATGGGCTCATATACCAAGGAGAAGTTCAAGCCCACTGAGATGGCAGCGG 3659
CILV-N      ACCCGGTTTACATGGGCTCGTATACCAAGGAGAAGTTCAAGCCCACTGAGATGGCGGCGG 3642
OFVRNA2     ATCCGGTTTATATGGGCTCATACACGAGGGAGAAGTTCAAGCCTACTGAGATGGCGGCGG 3642
            * ******** ******** ** ** * *************** *********** ****

CNSV        CTTACGGAGAGGAGGACCTGCTGTCCAGACCCATCCACCTGATGAAGCTGATCAATTGGA 3719
CILV-N      CTTACGGAGAGGAGGACCTACTGTCCAGACCCATCCACCTGATGAAGCTGATCAATTGGA 3702
OFVRNA2     CTTACGGAGAAGAGGACCTACTGTCCAGACCCATTCATCTGATGAAGCTAATCAATTGGA 3702
            ********** ******** ************** ** *********** **********

CNSV        GATATTCATCATCATCCAACATGGCCAGGATCCTCAAATGCCTGTTGAGCTCCCTGACTG 3779
CILV-N      GATACTCATCATCATCCAACATGGCCCGTACCCTCAAATGCCTGTTGAGCTCTCTGACTG 3762
OFVRNA2     GATATTCATCATCATCCAACATGGCCAGGATCCTGAAATGCCTGCTGAGCTCTCTTACTG 3762
            **** ********************* * * *** ********* ******* ** ****

CNSV        ATGCTGATGCTACACTTTTCTATGGGATGATGGAGTGGATTGCAGGGGATGCAGAGCATC 3839
CILV-N      ATGCTGATGCTACACTTTTCTATGGGATGATGGAGTGGATTGCAGGAGACGCAGAGCATA 3822
OFVRNA2     ATGCTGATGCCACTCTTTTCTACGGAATGATGGAGTGGATTGCAGGAGATGCAGAGCATA 3822
            ********** ** ******** ** ******************** ** *********

CNSV        GGTACCAGGATATGGCTACCAAACACGGGGGGGTCCCCAATGTTGCCCACTCTATACTGT 3899
CILV-N      GGTACCAGGACATGGCTACCAAACACGGGGGGGTTCCCAATGTCGCCCACTCTATACTGT 3882
OFVRNA2     GATATCAAGACATGGCGACCAAACATGGGGGAGTTCCCAATGTCGCTCACTCCATACTGT 3882
            * ** ** ** ***** ******** ***** ** ******** ** ***** *******

CNSV        CTTATGTCCGAGCTCACACCACCACATTCAGGAAGCATTCCCGAGGAGGGAAGAATGAGA 3959
CILV-N      CTTATGTCCGAGCTCACACCACCACATTCAGGAAGCATTCTCGAGGAGGGAAGAATGAGA 3942
OFVRNA2     CTTATGTCCGAGCTCACACCACCACATTTAGGAAACACTCCCGCGGAGGGAAAAATGAGA 3942
            **************************** ***** ** ** ** ******** *******

CNSV        CACTTCACTTCCAGGCTGTCATGATATACACATCAATGATAGCCCTGTTCAAGAAATACG 4019
CILV-N      CACTTCACTTCCAGGCCGTCATGATATACACATCAATGATAGCCCTGTTCAAGAAATACG 4002
OFVRNA2     CTCTCCACTTCCAGGCTGTCATGATATATACATCAATGATAGCCCTGTTCAAGAAATATG 4002
            * ** *********** *********** ***************************** *

CNSV        GGGGAATCATCCATTGGCATGAGAGCTGTGGAGAGTGCATCCAACAGACTGCTGGCAATG 4079
CILV-N      GGGGAATTATCCATTGGCATGAGAGCTGTGGAGAGTGCATCCAACAGACTGTCGGCAATG 4062
OFVRNA2     GGGGAATCATCCATTGGCATGAGAGCTGTGGCGAATGCATCCAGCAAACTGTGGGCAACG 4062
            ******* *********************** ** ******** ** ****  ***** *

CNSV        TGGATGTGGTCCTGACAAAGAGGGTGGAATTCCCAATATTGAAAGGAAATCTCTTTGCAT 4139
CILV-N      TGGATGTGGTCCTGACGAAGAGGGTGGAATTCCCAATATTGAAAGGAAATCTCTTTGCAT 4122
OFVRNA2     TTGATGTGGTACTGACAAAGAGGGTGGAGTTCCCGATCCTGAAAGGAAATCTCTTTGCAT 4122
            * ******** ***** *********** ***** **  *********************
```

```
CNSV        ATGTCCCGGCAGAAAGTATCAAATTCCATTATCATGACATGAAGAGGATACGGTCTGTGG 4199
CILV-N      ATGTCCCGGCAGAAAGTATCAAATTCCATTATCATGACATGAAGAGGATCCGGTCTGTGG 4182
OFVRNA2     ATGTCCCGGCGGAAAGTATAAAGTTTCACTACCATGACATGAAGAGGATTCGGTCTGTGG 4182
            ********** ******** ** ** ** ** ***************** **********

CNSV        AGGATATAGGATTCCGTACTGTGTCGTATGAATCTATGAGCTCCTTAGACAGAAGAACCG 4259
CILV-N      AGGATCTAGGATTCCGTACTGTGTCATATGAATCCATGAGCTCCTTAGACAGAAGAACCG 4242
OFVRNA2     AGGATCTGGGATTCCGTACTGTGTCATACGAATCCATGAGCCCCTTGGATAGAAGAACTG 4242
            ***** * ***************** ** ***** ****** **** ** ******** *

CNSV        CCAGCATCAGCATGCTGTCCGCAGTGGTATCAACGCAG--AGTACATGGGACAAGTCATC 4317
CILV-N      CCAGCATCAGCATGCTGTCCGCAGTGATATCATC-CAGTGAGTCCA-GGGACAAATCATC 4300
OFVRNA2     CCAGCATCAGCATGCTGTCCGCAGTGATATCGTC-CAGTGAGTCTA-GGGATAAATCATC 4300
            ************************** ****  * ***  ***  * **** ** *****

CNSV        AGTCACCATGAGTAGCATAGAAATGTGGGCCGGGACTGTCAATCTCACAGAGATCATCCC 4377
CILV-N      GGTTACCATGAGTAGCATAGAAATGTGGGCCGGGACTGTCAATCTCACAGAGATCATCCC 4360
OFVRNA2     TGTTACCATGAGTAGCATAGAGATGTGGGCCGGGACTGTCAATCTCACAGAGATCATCCC 4360
             ** ***************** **************************************

CNSV        AGCAGTGTGCCTGAGGGTGTCAGTGGCAGAGCTCATGAGAACAGGCGAATGGTCATGGCC 4437
CILV-N      CGCAGTGTGCCTGAGGGTGTCAGTGGCAGAGCTCATGAGAACAGGCGAATGGTCATGGCC 4420
OFVRNA2     TGCAGTATGCCTGAGGGTGTCGGTGGCCGAACTCATGAGGACGGGCGAATGGTCATGGCC 4420
             ***** ************** ***** ** ******** ** *****************

CNSV        TAAGCCTCACCTTAAGAAGCGAATAATCATGTTGTTAGAACCATTCATAAGGTCAAAGCT 4497
CILV-N      TAAGCCTCACCTTAAGAGGCGAATAATCATGTTATTAGAACCATTCATAAGGTCAAAGCT 4480
OFVRNA2     CAAGCCCCACCTCAAGAAAAGAATAATCATGCTACTAGAGCCATTCATAAGGTCAAAACT 4480
             ***** ***** ****   *********** *  **** ***************** **

CNSV        GGGGAGGTATCAGTTGTTGGAAATGGGTGTGACTCTGGATGAATTGCGATCCAACAATCC 4557
CILV-N      GGGGAGGTATCAGTTGTTGGAAATGGGTGTGACTCTGGATGAATTACGATCCAACAATCC 4540
OFVRNA2     TGGGAGGTACCAGCTGTTGGAAATGGGTGTGACCCTGGATGAACTACGATCCAACAACCC 4540
             ******** *** ******************* ********* * *********** **

CNSV        TGGAGTACTCCTGGACATCGTTCAGGTGGTCATGGACAACAGCAGGATGACACCGAAGAT 4617
CILV-N      TGGAGTGCTCCTGGATATCATTCAGGTAGTCATGGACAACAGCAGGATGACACCGAAGAT 4600
OFVRNA2     GGGGGTGCTCCTAGACATTGTTCAGGTGGTTATGGATAACAGCAGAATGACACCGAAAAT 4600
             ** ** ***** ** **  ******* ** ***** ******** *********** **

CNSV        CATCCCCAACAGAGTTTCCCCCAGCTGGGCTAACGATGTTATATGTGCTAAAATATATGA 4677
CILV-N      CATCCCCAATAGAGTTTCCCCCAGTTGGGCCAACGATGTTATATGTGCTAAGATATATGA 4660
OFVRNA2     TATCCCGAATAGAGTCTCTCCCAGTTGGGTCAATGACGTCATATGTGCTAAGATTTATGA 4660
             ***** ** ***** ** ***** ****  ** ** ** *********** ** *****

CNSV        GAATGAGCATATCATCTTCTGTTCCGGATGTTTGGCGAAAGGTCCATGTGTGGATAAAGT 4737
CILV-N      GAATGAGCATATCATCTTCTGTTCCGGATGTTTGGCGAAAGGTCCATGTGTGGATAAAGT 4720
OFVRNA2     GAATGAGCATATAATCTTCTGTTCCGGATGTCTGACAAAAGGCCCATGTGTGGATAAAGT 4720
            ************ ****************** ** * ***** *****************

CNSV        GGAGATATGCAATGATATCCTTATGGAATATACCTTCAAGGTGTGCTCAGCCACATTGCC 4797
CILV-N      GGAGATATGCAATGATATCCTTATGGAATACACCTTCAAGGTATGCTCAGCCACATTGCC 4780
OFVRNA2     GGAGATATGCAATGACATCCTCATGGAATACACCTTCAAGGTGTGTTCGGCTACATTGCC 4780
            *************** ***** ******** *********** ** ** ** ********

CNSV        GGTTCTCAAAGGGGACATGTCTCAGATCATATGTGATAGAGACTTCTCAAATGACCCGGA 4857
CILV-N      GGTCCTCAAAGGAGACATGTCTCAGATCATATGTGATAGAGACTTCTCAAATGACCCGGA 4840
OFVRNA2     AGTCCTCAAAGGAGACATGTCTCAGATTATATGTGATAGGGACTTCTCGAATGACCCAGA 4840
             ** ******** ************** *********** ******** ******** **

CNSV        TTGGACTTCATCGCCTGTCCAGTTGTTTGAAATGATGGACAATCCAGCTGCTCAGTTCAC 4917
CILV-N      TTGGACTTCATCGCCTGTCCAGTTGTTCGAAATGATGGACAATCCAGCTGCTCAGTTCAC 4900
OFVRNA2     TTGGACTTCATCGCCTGTCCAGTTGTTTGAAATGATGGACAATCCGGCTGCTCAATTCAC 4900
            *************************** ***************** ******** *****

CNSV        TCCATTCCCAAGGGACGGGCCCATAGCTGTGTCAAGGCCACTGGGCAGTGCCGGATTATC 4977
CILV-N      TCCATTCCCAAGGGACGGGCCCATAGCTGTGTCAAGGCCACTGGGCAGTGCCGGATTATC 4960
OFVRNA2     TCCATTCCCAAGGGACGGGCCCATAGCAGTGTCAAGACCATTGGGCAGTGCCGGATTGTC 4960
            *************************** ******** *** **************** **
```

```
CNSV       AAGCAGT---GGTATCAACGCAGAGTACAATGGGATTATCATGGTCGGGGATGACAGTGT 5034
CILV-N     AAGCATTCTGGATATACTGGCAGAGACCAATGGAATTATCATGGTCGGGGATGACAGTGT 5020
OFVRNA2    AAGCATCCTGGATATACTGGCAGAGACCAATGGAATCATCATGGTCGGGGATGACAGTGT 5020
           *****     * ***    ******  ****** ** ***********************

CNSV       CAGTATCACTATATGGAGACAGTTTAGAGCAACCAGGAGGGTTATGATCTTCAAGACAGG 5094
CILV-N     CAGTATCACTATATGGAGACAGTTCAGAGCAACCAGGAGGGTTATGATCTTCAAGACAGG 5080
OFVRNA2    CAGCATCACTATATGGAGACAGTTTAGAGCAACCAGGAAGGTGATGATTTTCAAGACAGG 5080
           *** ******************** ************* *** ***** ***********

CNSV       GGATAATCACCTTGAAGCTGCCTGTGACTTCATATGTATGGCCCCGGATGAAGCAGACCT 5154
CILV-N     GGATAATCACCTTGAAGCTGCCTGTGACTTCATATGTATGGCCCCTGATGAAGCAGACCT 5140
OFVRNA2    TGATAACCACCTTGAAGCTGCCTGTGACTTTATATGTATGGCCCCAGATGAAGCGGACCT 5140
            ***** *********************** ************** ******** *****

CNSV       ACTTATGGACAGTGAAAGTCCGGACGGGAAGGCTGATGTATATTACGGGGATAACATCCC 5214
CILV-N     ACTTATGGACAGTGAAAGTCCGGACGGGAAGGCTGATGTATATTATGGGGATAACATCCC 5200
OFVRNA2    GCTCATGGATAGTGAAAGCCCGGATGGGAAGGCTGACGTATATTATGGGGATAACATTCC 5200
            ** ***** ******** ***** *********** ******** *********** **

CNSV       TGTAGCTCCCGGTTGGTGGATAGTGACCAGCAGAAAGGATGTCAACAGATTGTACAAGTA 5274
CILV-N     TGTAGCTCCCGGTTGGTGGATAGTGACCAGTCGAAAAGATGTCAACAGATTGTACAAGTA 5260
OFVRNA2    TATAGCGCCCGGTTGGTGGATTATAACTAGCAGGAAAGATGTCAACAGATTGTACAAATA 5260
           * **** **************  * ** **  * ** ******************** **

CNSV       TGGTAAAGACATGAGCCCCACCAGGGTATTAATCTCCAAGGACAGAGGTCAAACCAGGGC 5334
CILV-N     TGGCAAAGACATGAGCCCCACCAGGGTATTAATCTCCAAGGACAGAGGTCAAACCAGGGC 5320
OFVRNA2    TGGGAAGGACATGAGCCCCACCAGGGTACTAATCTCCAAGGATAGAGGTCAAACCAGAGC 5320
           *** ** ********************* ************* ************** **

CNSV       AATCTGTGCTGTCAAGATGGAGCAATCCCCAGGCCAAAAGTGGGGAGACTTCCAAGCCAC 5394
CILV-N     AATCTGTGCTGTCAAGATGGAGCAATCCCCAGGACAAAAGTGGGGAGACTTCCAAGCCAC 5380
OFVRNA2    AATCTGTGCTGTCAAGATGGAGCAGACCCCGGGCCAGAAGTGGGGAGATTTTCAGGCCAC 5380
           ************************  **** ** ** *********** ** ** *****

CNSV       CTACAAAAAGATTGAGATGAGATGCAGGAACTACAGTGGGATGGTAAGAAATCCCACCAG 5454
CILV-N     CTACAAAAAGATTGAGATGAGATGCGGAAACTACAGTGGGATGGTAAGAAATCCCACCAG 5440
OFVRNA2    ATACAAGAAGATTGAAATGAGATGCGGAAACTACAGTGGGATGGTAAGAAATCCCACAAG 5440
            ***** ******** ********* * ***************************** **

CNSV       CAAGAACAAGTTAATATCATGCGCGATGTCATTATTCCACAGGGTCAATGGAGATATAGA 5514
CILV-N     CAAGAACAAGTTAGTATCATGCGCGGTGTCATTATTTCACAGGGTCAATGGAGATATAGA 5500
OFVRNA2    CAAGAACAAGTTAATATCATGTGCGGTGTCATTATTCCACAGGGTCAATGGAGATATAGA 5500
           ************* ******* *** ********** ***********************

CNSV       AGAGGCGGTCAAATGCTGTGTGACGGAGATGAGGACTATTTTCTGGAGGAACGAATTATA 5574
CILV-N     AGAGGCAGTCAAATGCTGTGTGACGGAGATGAGGACGATTTTCTGGAGGAACGAATTATA 5560
OFVRNA2    AGAGGCAGTCAAATGCTGTGTGACGGAGATGAGGACGATTTTCTGGAGGAACGAATTATA 5560
           ****** ***************************** ***********************

CNSV       TACTAAGAAGGCAAAGGAGCTGATGTATCTGATGTCGACACTGCACATGGCCGGCATGGA 5634
CILV-N     TACTAAGAAGGCAAAGGAGCTGATGTATCTGATGTCAACACTGCACATGGCCGGCATGGA 5620
OFVRNA2    TACTAAGAAAGCAAAGGATCTCATGTACCTGATGTCAACATTGCACATGGCAGGCATGGA 5620
           ********* ******** ** ***** ******** *** ********** ********

CNSV       GGGCGGAATAGAGTTTGCAAGACGATCTATCAAGCCTAGGGCAGTGAGGCGACCTTACAG 5694
CILV-N     GGGCGGAATAGAGTTTGCAAGACGATCTATCAAGCCTAGGGCAGTGAGGCGACCTTACAG 5680
OFVRNA2    GGGAGGGATAGAGTTTGCAAGACGATCTATCAAGCCTAGAGCAGTGAGACGACCTTACAG 5680
           *** ** ******************************** ******** ***********

CNSV       GATTCTCATCAAGACAACCGCTAGAAACAACTACAAGGCAGCTAATGATGTCACAGACGT 5754
CILV-N     GATTCTCATCAAGACAACCGCTAGAAACAACTACAAGGCAGCTAATGATGTCACAGACGT 5740
OFVRNA2    AATCCTCATCAAGACTACCGCCAGAAACAATTATAAGGCTGCCAATGATGTCACAGACGT 5740
            ** *********** ***** ******** ** ***** ** *****************

CNSV       GAGAGAGCTATTCAGAGTAACTCCCAAGCCTACCGGAGCACCCGGAGGAGATATTATCAT 5814
CILV-N     GAGAGAGCTATTCAGAGTAACTCCCAAGCCTACCGGAGCACCCGGAGGAGATATCATCAT 5800
OFVRNA2    GAGAGAGCTATTCAGGGTAACTCCCAAGCCTATCGGAGCACCCGGGGGAGATATCATCAT 5800
           *************** **************** ************ ******** *****
```

Cruz-Jaramillo *et al.*, 2014.

Trailer

```
CNSV       AAATATCGGGAAATAAGTATTTCTGTTTAAATTATATTCATTTAAGGAAAACCGGGTATC 5874
CILV-N     AAATATCGGGAAATAAGTAATTTTGTTTAAATTATATTCATTTAAGGAAAAA-------- 5852
OFVRNA2    AAATATCGGGAAATAAGTAATTTTGTTTAAATTATATACATTTAAAGAAAACCGGGTATC 5860
           ******************* ** ************** ******* *****

CNSV       TCGGGTTCCTCCC-ACAATCCTCCATCTCTGTTCACAACTGCGTTAAGAAGGACTAGGAC 5933
CILV-N     ---------------------------------------------AAAAAAAA------- 5860
OFVRNA2    TCGGGCTCCCCCCACTATCGTATATTTTTGTTCACAACTGCGTTAAGAAGGACTAGGAC 5920
                                                        ** **  *

CNSV       TAGGTCCCGAAGGTGCAACTGGGTCACACCGGAGTCGCAAGGAGATTTCATCAATCCATG 5993
CILV-N     ------------------------------------------------------------
OFVRNA2    TAGGTCCCGGAGGTGCAACTGGGTCACACCGGAGTCGCAAGGAGATTTCATCAATCCATG 5980

CNSV       TACTGTTGATCTGTCCTCTGCTTCC 6018
CILV-N     -------------------------
OFVRNA2    TACTGTTGATCTGTCCTGTGT---- 6001
```

Figure S4. Gene Ontology terms of upregulated and downregulated genes in CNSV-infected plants, according to RNA expression analyses. A graphic demonstration of genes with altered expression, classified in cell function, metabolism, secondary metabolites and biotic stress. Red, downregulated genes; blue, upregulated genes.

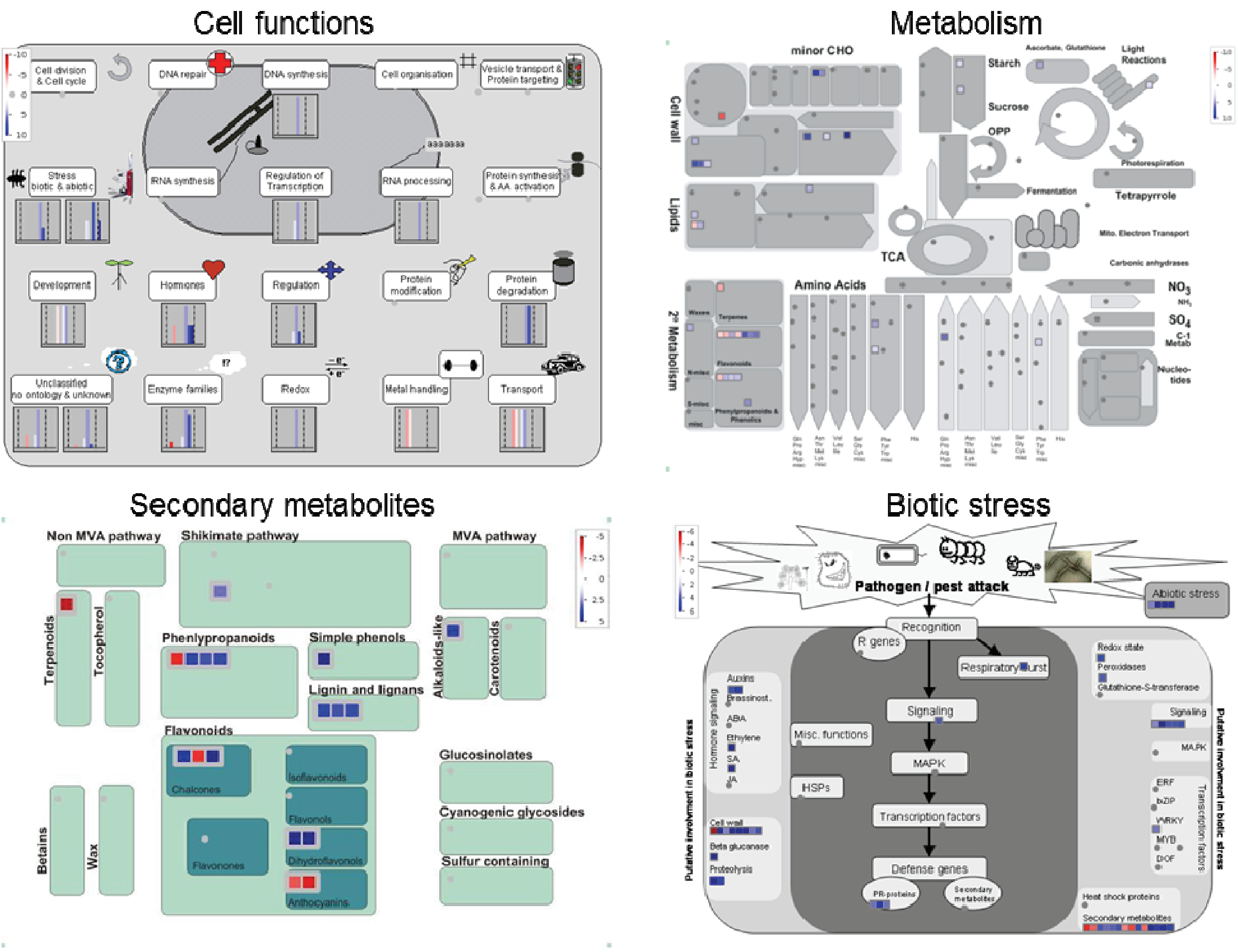

Table S1. Viral Operational Taxonomic Units (OTUs) used for phylogenetic reconstruction.

Abbreviation	Name	Description	Accession No.
LNYV	Lettuce Necrotic Yellows Virus	Cytorhabdovirus	Q86134.1 (N)<br>Q4W382.1 (L)
LYMV	Lettuce Yellow Mottle Virus	Cytorhabdovirus	NC_011532.1
NCMV	Northern Cereal Mosaic Virus	Cytorhabdovirus	NC_002251.1
CNSV	Citrus Necrotic Spot Virus	Dichorhavirus<br>**This work*	KF198064.2 (N)<br>KF198065.1 (L)
CiLV-N	Citrus Leprosis Virus Nuclear Type	Dichorhavirus	KF209275.2 (N)<br>KF209276.1 (L)
OFV	Orchid Fleck Virus	Dichorhavirus	NC_009608.1 (N)<br>AB244418.1 (L)
EMDV	Eggplant Mottled Dwarf Virus	Nucleorhabdovirus	KJ082087.1
MFSV	Maize Fine Streak Virus	Nucleorhabdovirus	NC_005974.1
MIMV	Maize Iranian Mosaic Virus	Nucleorhabdovirus	DQ186554.1
MMV	Maize Mosaic Virus	Nucleorhabdovirus	AY618418.1
PYDV	Potato Yellow Dwarf Virus	Nucleorhabdovirus	NC_016136.1
RYSV	Rice Yellow Stunt Virus	Nucleorhabdovirus	NC_003746.1
SYNV	Sonchus Yellow Net Virus	Nucleorhabdovirus	NC_001615.2
TVCV	Taro Vein Chlorosis Virus	Nucleorhabdovirus	NC_006942.1
CRV	Coffee Ringspot Virus	*Unclassified*<br>Rhabdovirus	KF812525.1 (N)<br>KF812526.1 (L)
LBVAV	Lettuce Big Vein Associated Virus	Varicosavirus<br>(External Group)	Q91QN9.1 (N)<br>Q8B0U2.1 (L)

Table S2. Oligonucleotides used for validation through qPCR of up- and down-regulated Citrus genes.

Oligonucleotide name	Sequence
Germin-like protein 5’	ATGGGAAGTTCTGCAAGGACCCCAAGCT
Germin-like protein 3’	CAACAGGATTTCACTGGCACGGGGGTGAAT
Thaumatin-like protein 5’	ATGAGGGAAGAGTACGCAAAACTGGACAA
Thaumatin-like protein 3’	ACAACAGCCCATTGAGGAAGCCCTCCAAAT
PR-3 class IV chitinase 5’	AGCCGAGCCAGGCACAACTAGAGCCCGCAT
PR-3 class IV chitinase 3’	ATCCGTCAATGTTGGACATGTCAATGAAAT
Proline-rich protein 4-like 5’	AGCAGATGCAGCACTTGATCCATCAGGGCT
Proline-rich protein 4-like 3’	TCCAGTGACAACTTTGAGGAGGAAGCTGGC
Aquaporin tip-2 like 5’	ATGGTTAGCCATGATTCCAGATTCGGAGCA
Aquaporin tip-2 like 3’	TGTATTTGATGAGGGTTTCCAGAATTCCAG
Cytochrome c oxidase 5’	GAGGCATTTTTGGATCACTTTT
Cytochrome c oxidase 3’	GTTCTGTTAGGTTCTTAGTAGC

Table S3. Upregulated genes in CNSV-infected Citrus.

	Length (bp)	Log2 fold-change
**Stress response and defense**		
Germin-like protein subfamily 1 member 14	898	11.3
1-aminocyclopropane-1-carboxylate synthase	1,534	9.8
Thaumatin-like protein	681	7.7
Blight-associated protein p12	483	7.2
PR-3 class IV chitinase	864	6.9
Stress-induced protein	213	6.9
Chalcone synthase	1,462	6.7
Beta- glucanase	1,190	7.2
Wound-induced protein win1	716	6.1
Cysteine-rich receptor-like protein kinase	1,869	6.0
Proline dehydrogenase mitochondrial-like	1,812	5.9
Blight-associated protein p12 precursor	632	5.8
Anthocyanidin 3-o-glucosyltransferase 5-like	1,434	5.8
Beta-glucosidase 41-like	1,894	5.8
Xylosyltransferase 1-like	1,293	5.8
Non-specific lipid-transfer protein at2g13820-like	958	5.7
Reticuline oxidase-like	1,617	5.6
Pathogenesis-related protein 1	480	5.5
Bifunctional nuclease I	941	5.5
Flavanone 3-beta-hydroxylase	1,403	5.4
GH3 family protein	2,138	5.4
Respiratory burst oxidase homolog protein b-like	2,855	5.3
Ribonuclease 3-like	925	5.3
Stress-response protein	694	5.3
Laccase 110	1,999	5.2
Methyl esterase	870	5.1
Auxin-induced protein	524	4.9
Serine protease inhibitor	285	4.8
2-oxoglutarate-dependent dioxygenase	1452	4.6
Beta –glucanase	1,404	4.5

Wound-induced protein WIN2	761	4.2
Quinone reductase family protein	853	4.2
Proteinase inhibitor	674	4.2

	**Length (bp)**	**Log2 fold-change**
**Stress response and defense** *(continued)*		
Phenylalanine ammonia-lyase	2,559	4.1
Stress-induced nucleic acid binding protein	696	4.1
Peroxidase 55-like	1,126	4.0
MLO-like protein 12	1,665	4.0
Monogalactosyldiacylglycerol synthase chloroplastic-like	1,890	3.9
Cinnamoyl- reductase	1,344	3.8
Zinc finger protein zat11-like	834	3.8
Protein TIFY 5a-like	882	3.8
Proline-rich protein	702	3.8
Thaumatin-like protein	928	3.8
Stress-induced homologue At5g58960	2,040	3.8
Cysteine proteinase	1,452	3.7
15-hydroxyprostaglandin dehydrogenase	857	3.7
Probable ribose-5-phosphate isomerase-like	2,316	3.7
Avr9 cf-9 rapidly elicited protein	1,646	3.6
TMV resistance protein n-like	3,014	3.5
Miraculin-like protein 2	1,099	3.5
Pathogenesis-related protein 4	771	3.5
Probable WRKY transcription factor 28-like	1,472	3.4
Protein TIFY 10[a]	1,081	3.4
Spx domain-containing protein 3-like	1,179	3.3
Cytochrome P450	1,493	3.2
Protein plant cadmium resistance 2-like	802	3.2
**Primary metabolism**		
Probable rhamnogalacturonate lyase b-like	2,303	10.5
Inositol oxygenase	1,246	8.8
Nucleotide binding protein	2,173	6.9

Udp-glycosyltransferase 74f2-like	1,401	6.5
Protein LURP-one-related 6-like	814	6.0
BAHD acyltransferase at5g47980-like	1,623	5.3
Inositol oxygenase 1	1,291	5.2
Sarcoplasmic reticulum His-rich calcium-binding	1,027	5.2
Polygalacturonase qrt3-like	1,545	5.0

	**Length (bp)**	**Log2 fold-change**
**Primary metabolism** *(continued)*		
Cysteine proteinase	1,326	4.9
Protein Ciclev10006313m	7,51	5.6
UDP-glycosyltransferase 74f2-like	1,401	4.7
Aminotransferase ALD1-like	1,386	4.7
NADPH-dependent codeinone reductase 2	1,307	4.6
Nucleotide-diphospho-sugar transferase family	1,080	4.6
Indole-3-acetic acid-amido synthetase	2,304	4.4
Tryptophan decarboxylase	1,592	4.3
Pectinesterase 2-like	1,722	4.3
Probable acyl-activating enzyme peroxisomal-like	1,689	4.2
Type I inositol- -trisphosphate 5-phosphatase 11	966	4.2
Bahd acyltransferase At5g47980-like	1,314	4.1
Potassium transporter 5-like	2,521	4.0
Alpha beta-hydrolases superfamily	1,628	3.6
Plant cell wall protein 88	681	3.6
4-coumarate: ligase	1,922	3.6
Xyloglucan endotransglucosylase hydrolase	762	3.5
Purple acid phosphatase 3 isoform 1	1,586	3.5
Beta- soluble isoenzyme i-like	2,310	3.4
Aminotransferase family protein	1,816	3.4
Kiwellin	753	3.3
Probable beta-d-xylosidase 2-like	2,604	3.3
Cdp-diacylglycerol--glycerol-3-phosphate 3-	573	3.3
Sulfate transporter -like	2,435	3.0

Alpha-amylase	1,481	3.0
Arogenate dehydratase prephenate dehydratase	1,756	2.9
Chlorophyll a/b binding protein	1,037	2.9
Hypothetical protein PRUPE_ppa013271mg	764	2.8
**Plant development**		
Zinc finger protein constans-like 16-like	1,976	3.2
Homeodomain-like superfamily isoform 1	2,662	2.8
**Signal transduction**		
Wall-associated receptor kinase-like protein	1,845	4.2
Probable LRR receptor-like protein kinase	2,351	4.2
Alpha beta-hydrolases superfamily protein	2,030	3.9
Probable LRR receptor-like Ser-Thr-protein kinase	3,195	3.2
Receptor like protein 1	2,210	4.5
**Proteins of unknown function**		
Uncharacterized protein TCM_007254	605	5.4
Hypothetical 21 kDa protein Ciclev10022285m	1,377	4.0
Uncharacterized protein LOC100782360 precursor	810	3.3
Uncharacterized protein TCM_034658	844	3.3

Table S4. Downregulated genes in CNSV infected Citrus.

	Length (bp)	Log_2 Fold Change
**Stress response and defense**		
Heavy metal transport detox. superfamily protein	1,136	-3.4
Protein aluminum sensitive 3-like	1,208	-3.0
Anthocyanin 5-aromatic	1,638	-4.2
Gibberellin 2-oxidase	1,253	-4.4
S-linalool synthase	2,763	-4.6
Aquaporin TIP2-1-like	1,117	-5.1
Proline-rich protein 4-like	1,384	-6.6
**Primary metabolism**		
BADH acyltransferase dcr-like	1,810	-3.8
NADH-dependent 6-deoxychalcone synthase-like	1,842	-3.3
Non-functional NADPH-dependent codeinone reduct.	922	-3.6
Respiratory burst oxidase homolog protein b-like	2,855	-3.7
Alpha beta fold family protein	1,119	-3.7
Plant-specific domain tigr01589 family protein	1,530	-5.4
**Proteins of unknown function**		
21 kDa protein Ciclev10005968m	1,105	-6.4

Table S5. Quantitative RT-PCR of five genes in virus-infected Citrus.

	Gene ID	Mass Fraction Symptomatic	Mass Fraction Asymptomatic	Fold Change	Standard Deviation	*p*-value	Statistical Significance
Upregulated*	Germin-like protein	17.0810	3.9332	4.34	0.3113	0.0112	yes
	Thaumatin-like protein	0.5848	0.3143	1.86	0.2071	0.0260	yes
	PR-3 class IV chitinase	7.7401	2.6710	2.90	0.1853	0.0441	yes
Downregulated*	Proline-rich protein 4-like	0.1783	0.2848	0.63	0.0384	0.0078	yes
	Aquaporin tip-2 like	0.0335	0.0700	0.48	0.0131	0.0011	yes
